# Elevated COUP-TFII expression in dopaminergic neurons accelerates the progression of Parkinson’s disease through mitochondrial dysfunction

**DOI:** 10.1371/journal.pgen.1008868

**Published:** 2020-06-24

**Authors:** Chung-Yang Kao, Mafei Xu, Leiming Wang, Shih-Chieh Lin, Hui-Ju Lee, Lita Duraine, Hugo J. Bellen, David S. Goldstein, Sophia Y. Tsai, Ming-Jer Tsai

**Affiliations:** 1 Department of Molecular and Cellular Biology, Baylor College of Medicine, Houston, Texas, United States of America; 2 Institute of Basic Medical Sciences, College of Medicine, National Cheng Kung University, Tainan, Taiwan; 3 Institute of Molecular Medicine, College of Medicine, National Cheng Kung University, Tainan, Taiwan; 4 Department of Physiology, College of Medicine, National Cheng Kung University, Tainan, Taiwan; 5 Department of Molecular and Human Genetics, Baylor College of Medicine, Houston, Texas, United States of America; 6 Howard Hughes Medical Institute, Baylor College of Medicine, Houston, Texas, United States of America; 7 Program in Developmental Biology, Baylor College of Medicine, Houston, Texas, United States of America; 8 Department of Neuroscience, Baylor College of Medicine, Houston, Texas, United States of America; 9 Jan and Dan Duncan Neurological Research Institute, Texas Children's Hospital, Houston, Texas, United States of America; 10 Clinical Neurocardiology Section, National Institute of Neurological Disorders and Stroke, National Institutes of Health, Bethesda, Maryland, United States of America; Montreal Neurological Institute, CANADA

## Abstract

Parkinson’s disease (PD) is a neurodegenerative disorder featuring progressive loss of midbrain dopaminergic (DA) neurons that leads to motor symptoms. The etiology and pathogenesis of PD are not clear. We found that expression of COUP-TFII, an orphan nuclear receptor, in DA neurons is upregulated in PD patients through the analysis of public datasets. We show here that through epigenetic regulation, COUP-TFII contributes to oxidative stress, suggesting that COUP-TFII may play a role in PD pathogenesis. Elevated COUP-TFII expression specifically in DA neurons evokes DA neuronal loss in mice and accelerates the progression of phenotypes in a PD mouse model, MitoPark. Compared to control mice, those with elevated COUP-TFII expression displayed reduced cristae in mitochondria and enhanced cellular electron-dense vacuoles in the substantia nigra pars compacta. Mechanistically, we found that overexpression of COUP-TFII disturbs mitochondrial pathways, resulting in mitochondrial dysfunction. In particular, there is repressed expression of genes encoding cytosolic aldehyde dehydrogenases, which could enhance oxidative stress and interfere with mitochondrial function via 3,4-dihydroxyphenylacetaldehyde (DOPAL) buildup in DA neurons. Importantly, under-expression of COUP-TFII in DA neurons slowed the deterioration in motor functions of MitoPark mice. Taken together, our results suggest that COUP-TFII may be an important contributor to PD development and a potential therapeutic target.

## Author summary

Parkinson’s disease (PD) is one of the major neurodegenerative diseases with increased risks in the elderly population. The patients mainly experience motor dysfunction, such as tremors, bradykinesia, rigid muscles, and impaired posture and balance. However, the underlying cause of PD is poorly defined in most cases. Understanding the etiology of PD will help prevention or treatment of this disease. Here, we reported that a transcription factor, COUP-TFII, is upregulated in the substantia nigra and dopaminergic neurons (DAs) of PD patients. Elevated expression of COUP-TFII leads to aberrant cell death of DAs, a group of neurons in the central nervous system that controls body movement. The underlying mechanism is enhanced oxidative stress due to COUP-TFII-mediated transcriptional repression of mitochondrial proteins for energy production and enzymes for detoxifying dopamine metabolites. Therefore, targeting COUP-TFII in dopaminergic neurons may be a potential therapeutic strategy for PD.

## Introduction

Parkinson’s disease (PD) is one of the major neurodegenerative diseases in the United States [1]. Its prevalence is about 1% of the population over 65 years old [2]. PD is known to cause progressive cell loss in various regions of the brain [3]. The cell loss of dopaminergic (DA) neurons in the substantia nigra (SN) results in insufficient dopamine in the striatum. Reduction of dopamine, which is responsible for transmitting signals within the brain to coordinate movement, leads to motor dysfunction, such as tremors, bradykinesia, rigid muscles, and impaired posture and balance. Currently, there is no cure for PD. L-DOPA, a dopamine precursor, is the standard treatment for PD, although it can induce adverse complications and resistance [4].

The etiology of PD remains largely undefined since only 10% of the patients have a family history, whereas nearly 90% of PD patients are considered sporadic [5]. Gene mutations and environmental toxins have been associated with the causes of PD. Direct evidence from familial PD has shown that individuals with multiple copies of *SNCA* or disruption of the PINK1/PARKIN axis may develop early-onset PD [6,7]. Epidemiological studies demonstrated that long-term exposure to pesticides or drugs, such as rotenone and MPTP (1-methyl-4-phenyl-1,2,3,6-tetrahydropyridine), increases the risk of developing PD [8]. Recently, emerging evidence indicated that mitochondrial dysfunction contributes to the pathogenesis of PD [9]. Mutations of PINK1 or PARKIN give rise to poor mitochondrial quality control in the clearance of damaged mitochondria, which is also known as mitophagy. Moreover, rotenone and MPTP can induce neurotoxicity through the inhibition of the mitochondrial respiratory chain complex I [10]. Accumulation of damaged mitochondria and complex I inhibition is believed to cause the formation of toxic levels of reactive oxygen species (ROS), eventually leading to cell death of DA neurons [11–13].

Chicken ovalbumin upstream promoter-transcription factor II (COUP-TFII also known as NR2F2) is an orphan nuclear receptor. The expression of COUP-TFII is abundant in the embryonic stages but gradually decreases during development. Our group and others have shown that COUP-TFII plays critical roles in cardiovascular development, neuronal development, organogenesis, reproduction, and metabolism [14]. Although COUP-TFII has been considered as a gene important for development, our studies have recently uncovered its pathological roles in adulthood. Elevated COUP-TFII expression has been implicated to play a role in the pathogenesis of the following diseases, including prostate cancer, heart failure, and muscular dystrophy [15–18]. We demonstrated that in adult mouse hearts, elevated COUP-TFII expression could preferentially repress genes important for mitochondrial electron transport chain enzymatic activities, oxidative stress detoxification, and mitochondrial dynamics [16]. The reduction of gene expression essential for the maintenance of a healthy and functional mitochondrial network by COUP-TFII resulted in mitochondrial dysfunction and increased ROS production, leading to the development of dilated cardiomyopathy.

In sporadic PD patients, dysregulated networks of mitochondrial pathways and autophagic degradation systems have been identified based on genome-wide gene expression profiling analyses of postmortem brain tissues [9]. Genome-wide association studies (GWAS) also identified numerous susceptible loci [19,20], indicating that in addition to environmental toxins, unknown genetic predisposition can be one of the causal factors involved in the etiology and pathogenesis of sporadic PD. Nevertheless, PD, especially sporadic PD, is considered as the outcome of a complex dysregulation of gene networks contributed by multiple factors [21]. In order to slow down the progressive neurodegeneration, it is important to find the key contributors or modifiers in the aforementioned pathways that contribute to the development of sporadic PD. Since COUP-TFII has been demonstrated as a master regulator in the mitochondrial pathways in adult hearts, we speculated that COUP-TFII might also play a role in the pathogenesis of sporadic PD. We found that COUP-TFII expression is upregulated in the DA neurons of PD patients, implicating COUP-TFII as a potential player in the pathogenesis of PD. To test this hypothesis, we utilized mouse models overexpressing COUP-TFII to investigate the functions of COUP-TFII in midbrain DA neurons.

## Results

### COUP-TFII is upregulated in PD patients, and oxidative stress induces COUP-TFII expression through epigenetic regulation

**Fig 1:**
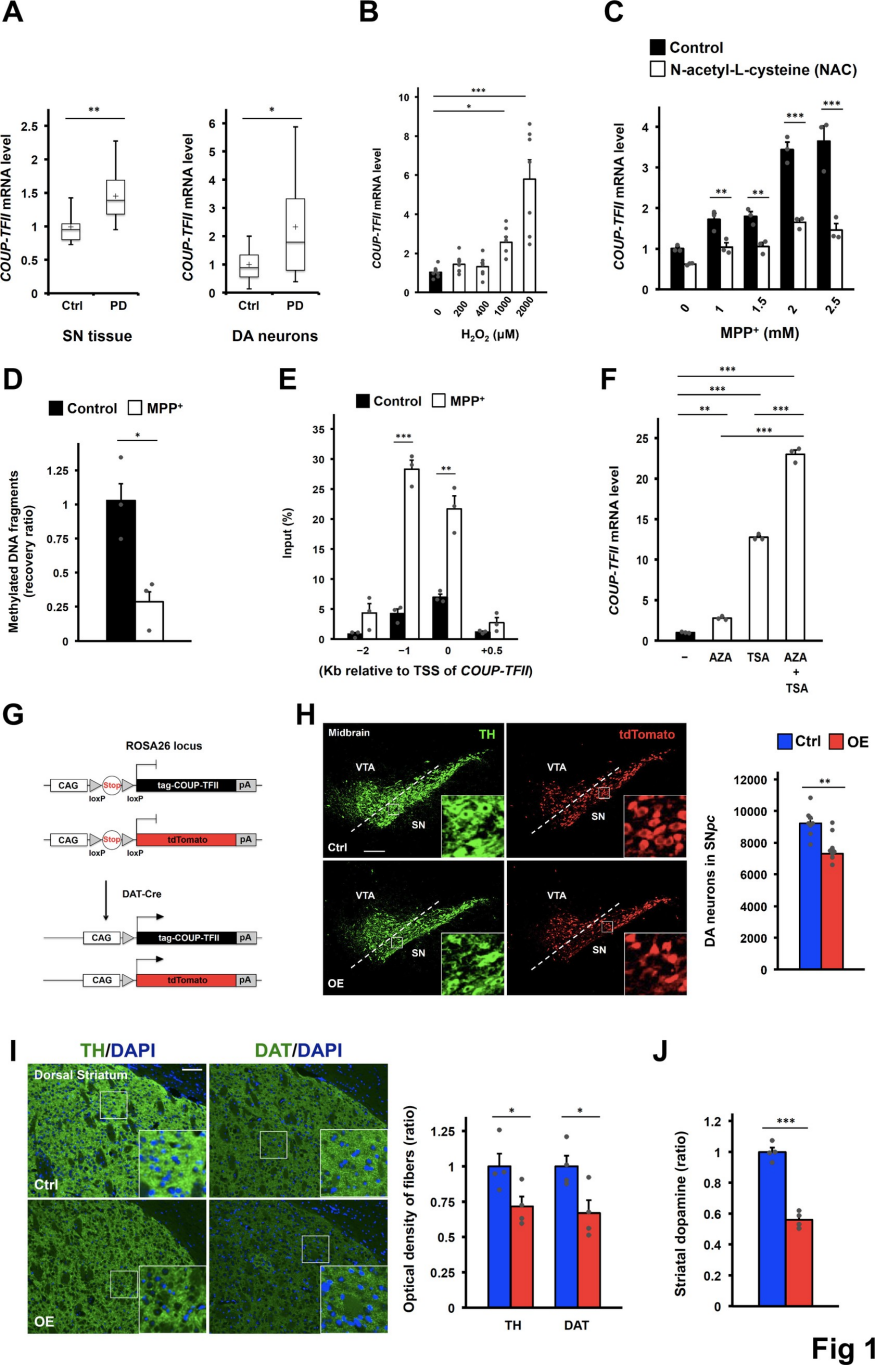
Elevated COUP-TFII expression in DA neurons causes mild degeneration in mice. **(A)** COUP-TFII mRNA levels in the substantia nigra (SN) tissues (left) of healthy controls (n = 9) and sporadic PD patients (n = 16) from GSE7621 and in DA neurons (right) of the SN tissues from healthy controls (n = 8) and sporadic PD patients (n = 10) from GSE20141. **(B)** COUP-TFII mRNA levels in the differentiated SH-SY5Y cell line after 24-hour H_2_O_2_ treatment. n = 7/group from repeated independent experiments. One-way ANOVA Fisher’s LSD post hoc test. **(C)** COUP-TFII mRNA levels in the differentiated SH-SY5Y cell line after 24-hour neurotoxin MPP^+^ treatment in the absence or presence of 2 mM N-acetyl-L-cytsteine (NAC) antioxidant. n = 3 replicates/group. Two-way ANOVA Fisher’s LSD post hoc test. This experiment was independently repeated and produced with similar results. **(D)** Relative recovery ratio of methylated DNA fragments from the gene locus of COUP-TFII in the differentiated SH-SY5Y cell line after 24-hour neurotoxin MPP^+^ (1mM) treatment. n = 3/group. **(E)** ChIP-qPCR results of histone H3 acetylation at the gene locus of COUP-TFII in the differentiated SH-SY5Y cell line after 24-hour neurotoxin MPP^+^ (1mM) treatment. TSS, transcription start site. n = 3/group. **(F)** COUP-TFII mRNA levels in the differentiated SH-SY5Y cell line after 4-day DNA methyltransferase inhibitor (1 μM, AZA) and/or 1-day histone deacetylase inhibitor (0.5 μM, TSA) treatment. AZA, 5-aza-2'-deoxycytidine; TSA, Trichostatin A. n = 3/group. Two-way ANOVA Fisher’s LSD post hoc test. **(G)** Strategy to overexpress COUP-TFII in DA neurons in mice. DAT-Cre, Cre recombinase expression under the control of the endogenous dopamine transporter (DAT) gene; tag, MYC-Tag; CAG, CAG promoter; pA, poly(A) signals. **(H)** Representative images (left) and quantification (right) of DA neurons in the ventral midbrain of 16- to 17-week-old control (Ctrl) and COUP-TFII overexpression (OE) mice after staining with anti-tyrosine hydroxylase (TH) or anti-tdTomato antibodies (higher-power views in the insets). n = 8/group. Scale bar, 200 μm. **(I)** Representative images (left) and quantification (right) of DA axonal projections to the dorsal striatum of 16- to 17-week-old mice after staining with anti-TH or anti-DAT antibodies (higher-power views in the insets). n = 4/group. Scale bar, 100 μm. **(J)** Relative total striatal dopamine of 16- to 17-week-old mice. n = 4/group. **(A-J)** *p < 0.05; **p < 0.01; ***p < 0.001 compared to control (*t*-test if not indicated). Mean ± SEM. See also S1 Fig.

To examine whether there is an association between COUP-TFII and PD, we first utilized public datasets to analyze COUP-TFII expression in PD patients, in induced differentiation of pluripotent stem (iPS)-derived DA cells, and in MPTP-treated mice. We found that COUP-TFII expression levels were significantly elevated in two independent cohorts of sporadic PD patients (Fig 1A). In one dataset, COUP-TFII expression levels were shown to be upregulated by 1.5-fold in the SN tissues of sporadic PD patients (GSE7621) [22]. The SN tissues of postmortem PD and non-PD subjects were collected from frozen coronal blocks based on surface and cytoarchitectural landmarks [23]. In the other dataset, COUP-TFII expression levels were shown to be upregulated by 2.3-fold in DA neurons isolated by laser capture microdissection from sporadic PD patients (GSE20141) [24]. About 80 to 200 darkly pigmented neurons were collected from the SN *pars compacta* (SN*pc*) in cryosections of postmortem PD and non-PD subjects. Moreover, iPS cells differentiated into DA neurons from familial PD patients with a mutated SNCA (SNCA-A53T) resulted in an increase of COUP-TFII expression (S1A Fig) [25]. In addition to the models of genome-based mutations, mice treated with a neurotoxin prodrug, MPTP, exhibited increased levels of COUP-TFII in the SN tissues and in isolated DA neurons (S1B Fig) [26,27]. These data indicate that not only sporadic PD but also familial or chemical-induced PD models can induce COUP-TFII expression.

Since oxidative stress has long been implicated in PD [11–13], we wondered whether ROS can directly induce COUP-TFII expression. Indeed, when the differentiated DA neuron-like neuroblastoma SH-SY5Y cells were treated with hydrogen peroxide (H_2_O_2_), COUP-TFII expression was induced in a dose-dependent manner (Fig 1B). Furthermore, when cells were treated with MPP^+^ (the active metabolite of the electron transport complex I inhibitor MPTP) or environmental toxins (rotenone and paraquat), enhanced expression of COUP-TFII was similarly observed (S1C Fig). The increase in COUP-TFII expression was diminished when the cells were co-cultured with the antioxidant N-acetyl-L-cysteine (NAC) and MPP^+^ (Fig 1C). Since it has been reported that overexpressing SNCA induces ROS [28], SY-SH5Y cells overexpressing SNCA-WT or SNCA-A53T was established to examine whether COUP-TFII expression can be induced in a different PD model. Indeed, COUP-TFII expression was induced in these overexpression cells, and the increased expression was reversed subsequent to NAC treatment (S1D Fig). It is known that oxidative stress may affect gene expression through epigenetic regulation [29]. To gain insight into how COUP-TFII is regulated in PD, DNA methylation and histone acetylation were examined at the gene locus of COUP-TFII. Decreased methylation of CpG islands (Fig 1D and S1E Fig) and increased histone acetylation (Fig 1E and S1E Fig) near the proximal promoter region of COUP-TFII were detected in the cells treated with MPP^+^, suggesting that COUP-TFII expression is regulated by epigenetic modification. Treatment with DNA methyltransferase inhibitor (AZA) and histone deacetylase inhibitor (TSA) by itself or together enhanced the expression of COUP-TFII (Fig 1F), further confirming the importance of epigenetic regulation. These results indicate that oxidative stress is associated with elevated COUP-TFII expression.

### Mice with elevated COUP-TFII expression in DA neurons of the SN*pc* display mild neurodegeneration

Although elevated COUP-TFII expression was observed in PD patients and the mouse model, the main question is whether it contributes to neurodegeneration. For this purpose, we generated a mouse model ectopically expressing COUP-TFII specifically in DA neurons to investigate the potential link between PD and COUP-TFII upregulation (Fig 1G). In addition, we expressed the tdTomato reporter under the control of the CAG promoter specifically in DA neurons to facilitate unbiased counting of DA neurons. In this mouse model, the levels of COUP-TFII mRNA were overexpressed about 2-fold (S1F Fig), similar to levels seen in PD patients. Elevated COUP-TFII protein in the midbrain was shown to colocalize only with tyrosine hydroxylase^+^ (TH^+^) positive cells (S1G Fig). Together, these results indicate that this mouse model can serve as a good tool to study the roles of COUP-TFII specifically in DA neurons.

At 16–17 weeks of age, mice overexpressing COUP-TFII (OE mice) displayed a small reduction in the number of DA neurons relative to control (Ctrl) mice, as assessed by immunostaining of TH or the tdTomato reporter (Fig 1H). The results of stereological neuron counting estimated a 15–20% decrease in tdTomato^+^ neurons within the SN*pc*. This result also indicated that the loss of DA neurons was due to cell death instead of the sensitivity of TH immunostaining. Moreover, DA axonal projections to the dorsal striatum of OE mice exhibited a 25–35% reduction in the density of axonal innervation as determined by different DA molecular markers (Fig 1I and S1F Fig). In agreement with the density of axonal innervation, a similar reduction in total striatal dopamine was observed in OE mice at 16- to 17-weeks old (Fig 1J). We also examined mice with an age of 1.5 years. However, there was no further significant decrease in dopamine levels between young and aged OE mice (S1H Fig). This level of decrease in dopamine levels did not result in motor dysfunction as expected (S1I Fig). These findings indicate that elevated COUP-TFII expression alone can cause mild degeneration of DA neurons, but is not sufficient to lead to severe PD phenotypes.

### Elevated COUP-TFII expression contributes to progressive neurodegeneration of PD in a mouse model of mitochondrial dysfunction

Sporadic PD is a complex disorder caused by multiple genetic and environmental factors [21]. In order to examine whether elevated COUP-TFII expression can contribute to the disease progression of PD, we crossed OE mice with MitoPark mice, a progressive PD mouse model with impaired respiratory chain function in DA neurons [30]. In the early stages of MitoPark mice, methylation of CpG islands in the COUP-TFII locus of the SN*pc* was reduced (S2A Fig), which may contribute to elevated expression of COUP-TFII in the SN*pc* (S2B Fig). Ctrl, OE, MitoPark, and MitoPark/OE mice were born at the expected Mendelian ratios and had normal gross appearance after birth. However, MitoPark/OE mice started to show ill appearance and significantly reduced body weight at 24 weeks of age (S2C Fig). In agreement with previous reports, MitoPark mice displayed premature death (median survival time of 254 days), whereas MitoPark/OE mice had an even shorter median survival time (232 days) (S2D Fig). These observations suggest that elevated COUP-TFII expression accelerates PD phenotypes of MitoPark mice.

**Fig 2:**
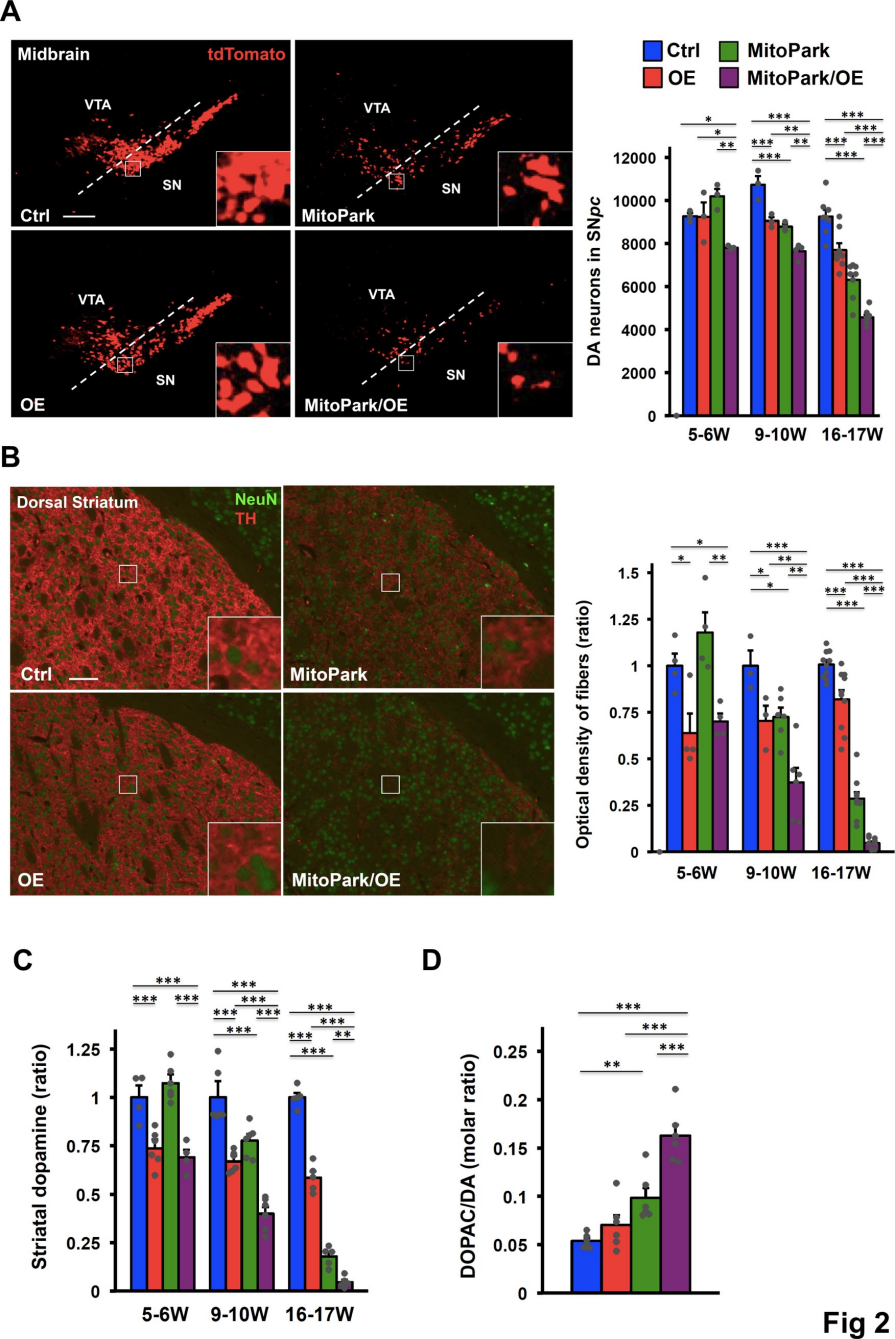
Elevated COUP-TFII expression contributes to progressive neurodegeneration of MitoPark mice. **(A)** Representative images (left) of DA neurons in the ventral midbrain of 16- to 17-week-old Ctrl, OE, MitoPark, and MitoPark/OE mice detected by the tdTomato reporter and quantification (right) of DA neurons in the SN*pc* of mice at 5–6 (n = 3/group), 9–10 (n = 3-4/group), and 16–17 (n = 8/group) weeks of age (higher-power views in the insets). Scale bar, 200 μm. **(B)** Representative images (left) of DA axonal projections to the dorsal striatum of 16- to 17-week-old mice after double staining with anti-TH and anti-NeuN antibodies and quantification (right) of TH^+^ fibers of mice at 5–6 (n = 4/group), 9–10 (n = 3 or 6/group), and 16–17 (n = 10/group) weeks of age (higher-power views in the insets). Scale bar, 100 μm. **(C)** Relative total striatal dopamine levels of mice at 5–6, 9–10, and 16–17 weeks of age. n = 4-6/group. **(D)** Dopamine turnover, as measured by the ratio of the DA metabolite 3,4-dihydroxypheylacetic acid (DOPAC) to DA, in 9- to 10-week-old mice. n = 6/group. **(A-D)** *p < 0.05; **p < 0.01; ***p < 0.001. Mean ± SEM. One-way ANOVA Fisher’s LSD post hoc test. See also S2 Fig.

DA neurons of these mice were examined at different time points before they began to show gross weakness. As reported, MitoPark mice displayed a slow progressive loss of DA neurons in the SN*pc* (Fig 2A). Overexpression of COUP-TFII in the MitoPark background accelerated the degeneration during the time points we analyzed. At 5–6 weeks of age, Ctrl, OE, and MitoPark mice had comparable numbers of DA neurons in the SN*pc*, while MitoPark/OE exhibited about a 20% reduction in DA neurons, indicating that elevated COUP-TFII expression contributes to the early initiation of the loss of DA neurons in the genetic background of MitoPark mice. The progressive loss of DA innervation was also observed in the dorsal striatum (Fig 2B). Consistently, axonal degeneration was more severe in MitoPark/OE mice as compared to MitoPark mice (S2E Fig). Notably, the loss of axonal innervation in the dorsal striatum was more prominent than the loss of DA neurons in the SN*pc* in both MitoPark and MitoPark/OE mice at the corresponding time points. In addition, OE mice had reduced TH^+^ fibers in the dorsal striatum without the loss of DA neurons at 5–6 weeks of age (Fig 2A and 2B). This phenotype suggests that the pathology in OE mice started at the DA neuron terminals, leading subsequently to the retrograde loss of cell bodies. In contrast, the ventral tegmental area (VTA), the other cluster of DA neurons adjacent to the SN tissue in the midbrain, was minimally affected by the overexpression of COUP-TFII (S2F Fig). Similarly, OE and MitoPark mice exhibited a mild loss of DA innervation in the ventral striatum, the brain nucleus of DA axonal projections from the VTA (S2G Fig). However, MitoPark/OE mice displayed a slight but significantly greater reduction in the optical density of TH^+^ axonal projections in the ventral striatum (S2H Fig). Overall, this phenomenon is consistent with the clinical observation that DA neurons in the VTA are less susceptible to degeneration than those in the SN*pc*.

Since the substantial loss of dopamine is the key feature of PD, the concentration of total striatal dopamine was determined (Fig 2C). The results matched the progressive loss of DA axonal projections. Next, we asked whether increased turnover of dopamine in PD patients due to compensatory mechanisms [31,32] could also be observed in our mouse model. At 9–10 weeks of age, when dopamine levels in MitoPark/OE mice were drastically reduced, we found an increase in dopamine turnover, as measured by the ratio of the dopamine metabolite 3,4-dihyroxyphenylacetic acid (DOPAC) to DA, and the upregulation of serotonin in MitoPark/OE mice (Fig 2D and S2F Fig), indicating that compensatory mechanisms due to dopamine consumption and axonal degeneration do occur in our mouse model.

### Elevated COUP-TFII expression accelerates motor symptoms of MitoPark mice, which can be reversed by L-DOPA treatment

**Fig 3:**
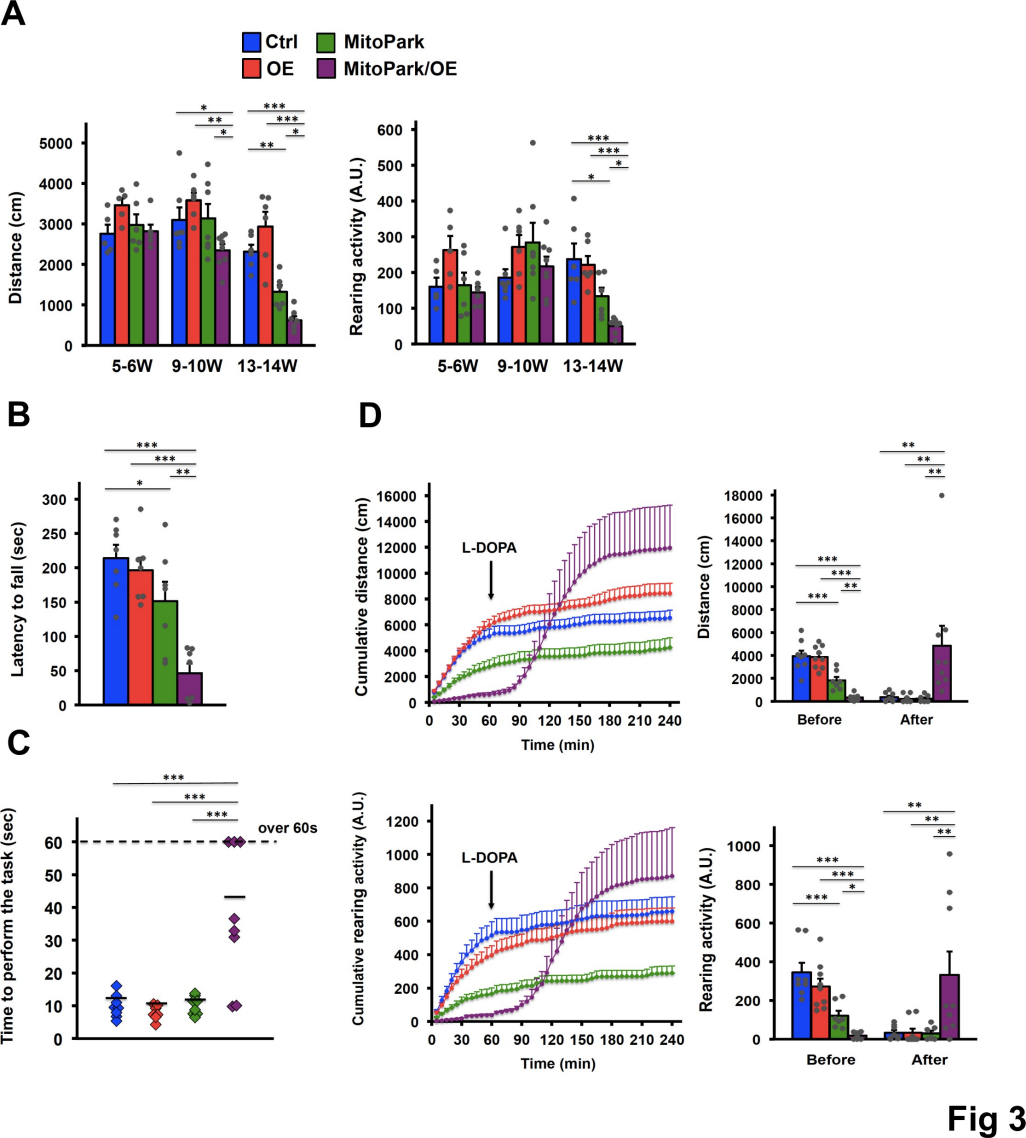
Accelerated motor symptoms of MitoPark mice by elevated COUP-TFII expression, which can be reversed by L-DOPA treatment. **(A)** Open-field assay recorded for Ctrl, OE, MitoPark, and MitoPark/OE mice at 5–6, 9–10, and 13–14 weeks of age. n = 5-8/group. **(B)** Rotarod performance test recorded for 15- to 18-week-old mice. n = 7/group. **(C)** Pole test recorded for 12- to 15-week-old mice. n = 7-8/group. **(D)** Open-field assay recorded for 15- to 16-week-old mice treated with L-DOPA (20 mg/kg). The arrows indicate reagent addition steps. Before, averaged activity from 0 to 30 minutes (pre-L-DOPA treatment); After, averaged activity from 75 to 105 minutes (post-L-DOPA treatment). n = 7-9/group. **(A-D)** *p < 0.05; **p < 0.01; ***p < 0.001. Mean ± SEM. One-way ANOVA Fisher’s LSD post hoc test. See also S3 Fig.

Dopamine insufficiency has been shown to give rise to motor dysfunction. Thus, we tested if the accelerated loss of dopamine in the genetic background of MitoPark mice with elevated COUP-TFII expression could cause neurobehavioral changes. The results showed that elevated COUP-TFII expression accelerated the defects of spontaneous locomotion activity in an open-field assay as measured by walking distance and rearing activity of 9- to 14-week-old mice (Fig 3A). For younger mice at 9–10 weeks of age, the loss of about 25% dopamine in OE and MitoPark mice was insufficient to cause motor dysfunction as expected (Fig 3A). In contrast, MitoPark/OE mice with more than 50% loss of dopamine levels at older ages started to exhibit shorter walking distances (horizontal activity) as compared to the other groups and worsened at 13–14 weeks of age. Also, MitoPark/OE mice had more impairment in rearing activity (vertical activity), which is the frequency of the mice standing up and exploring their surroundings, as compared to MitoPark mice at 13–14 weeks of age. Similarly, rotarod performance and pole tests, which are designed to specifically evaluate motor coordination, indicate that MitoPark/OE stayed for a shorter period on the accelerating rolling rod than MitoPark mice (Fig 3B) and required significantly more time to descend from the top of the pole to the ground (Fig 3C). These results indicate that elevated COUP-TFII expression in the genetic background of MitoPark mice not only leads to the extra loss of DA neurons, but is also sufficient to cause early-onset motor symptoms.

In order to examine whether motor symptoms were due to the defects in the nigrostriatal pathway, we treated MitoPark/OE mice with L-DOPA, a dopamine precursor used to ameliorate motor symptoms in PD patients. At 15–16 weeks of age, L-DOPA administration fully rescued the hypoactivity of MitoPark/OE mice assessed by open-field assay (Fig 3D and S3A Fig). In contrast, MitoPark mice at 15–16 weeks of age were less sensitive to L-DOPA (Fig 3D) since total striatal dopamine in MitoPark mice was only reduced to 20% of normal levels (Fig 2C). This is probably due to autoregulation of DA levels by presynaptic autoreceptors from the presence of enough amounts of healthy DA neurons [33]. The above observation is consistent with the previous report [34], demonstrating that the response of MitoPark mice to L-DOPA is more pronounced or overcompensated when total striatal dopamine is reduced to 10% of normal levels at 24 weeks of age. However, at 23–24 weeks of age, MitoPark mice started to show greater responsiveness to L-DOPA treatment than MitoPark/OE mice (S3B Fig). It is speculated that in late stages of PD patients, decreasing DA neurons results in fewer and fewer axon terminals capable of uptaking exogenous L-DOPA for subsequent efficient use [35], suggesting that overall phenotypes of MitoPark/OE mice at the time point analyzed are much worse than those in MitoPark mice. Collectively, the results indicate that accelerated motor dysfunction caused by elevated COUP-TFII expression is specifically due to the defects in the nigrostriatal pathway.

### Impaired mitochondrial pathways and enhanced oxidative stress in DA neurons with elevated COUP-TFII expression

**Fig 4:**
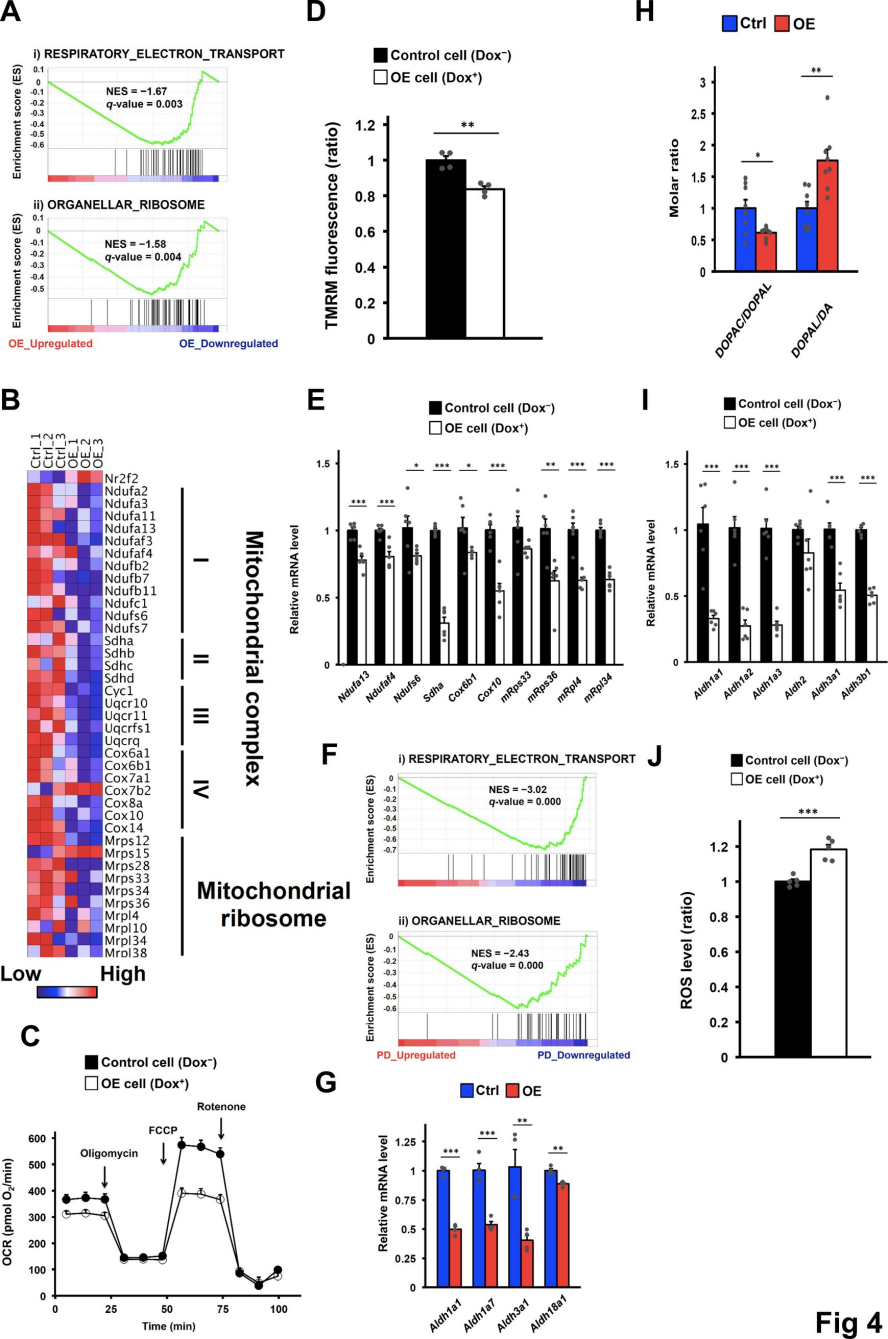
Elevated COUP-TFII expression impairs mitochondrial pathways and enhances oxidative stress in DA neurons. **(A)** Mitochondrial function-related gene signatures identified by GSEA in the transcriptional profiles from the ventral midbrain of 2-month-old Ctrl and OE mice. **(B)** Expression profile of affected mitochondrial essential genes from the ventral midbrain of 2-month-old Ctrl and OE mice. **(C)** Mitochondria oxygen consumption rates in the N27 rat DA neural cell line. The arrows indicate reagent addition steps. n = 10/group from repeated independent experiments. **(D)** Relative mitochondrial membrane potential in the N27 rat DA neural cell line. n = 4/group. **(E)** mRNA levels of key genes in mitochondrial pathways in the N27 rat DA neural cell line. n = 6/group. **(F)** Mitochondrial function-related gene signatures identified by GSEA from a publicly available human sporadic PD dataset (GSE20141). **(G)** Expression profile of affected ALDH genes in the ventral midbrain of 2-month-old Ctrl and OE mice. **(H)** Relative ratios of DOPAC to DOPAL or DOPAL to DA in the striatum of 2-month-old Ctrl and OE mice. n = 8/group. **(I)** mRNA levels of ALDHs in the N27 rat DA neural cell line. n = 6/group. **(J)** Relative cellular ROS levels in the N27 rat DA neural cell line. n = 5/group. **(A-J)** NES, normalized enrichment score; OCR, oxygen consumption rate; TMRM, tetramethylrhodamine methyl ester. *p < 0.05; **p < 0.01; ***p < 0.001 compared to control (*t*-test). Mean ± SEM. See also S4 Fig.

To gain insights into the impact of COUP-TFII on DA neurons, we profiled the molecular changes of 2-month-old Ctrl and OE mice through RNAseq analysis of the ventral midbrain (S1 Table). Gene ontology analysis revealed that downregulated genes in OE mice were enriched in the category of “Parkinson’s disease” in the Kyoto Encyclopedia of Genes and Genomes (KEGG) (S4A Fig). “Metabolic pathways” and “Oxidative phosphorylation” were also listed in the top 5 categories of COUP-TFII-downregulated genes, which is in line with our previous report on heart failure [16]. Furthermore, Gene Set Enrichment Analysis (GSEA) identified gene signatures of “Respiratory_Electron_Transport” and “Organellar_Ribosome” which are significantly associated with COUP-TFII-downregulated genes (Fig 4A), suggesting that elevated COUP-TFII expression represses genes required for the maintenance of mitochondrial function. Indeed, many genes in the mitochondrial respiratory chain complex and mitochondrial ribosome were repressed in OE mice (Fig 4B). Since DA neurons are intermixed with glutamatergic neurons, GABAergic neurons and glial cells in the ventral midbrain [36], in order to test whether elevated COUP-TFII expression affects mitochondrial function specifically in DA neurons, we overexpressed COUP-TFII using doxycycline-inducible systems in the N27 rat DA neural cell line. Oxygen consumption rate, an indicator of mitochondrial function, was monitored (Fig 4C). Maximal respiration and spare capacity were significantly reduced in COUP-TFII-overexpressing cells compared to control cells (Fig 4C and S4B Fig). Moreover, mitochondrial membrane potential was decreased in COUP-TFII-overexpressing cells (Fig 4D). However, mitochondrial DNA was significantly increased in COUP-TFII-overexpressing cells (S4C Fig), suggesting that compensatory mechanisms are inadequate to revert the phenotypes of mitochondrial dysfunction exerted by elevated COUP-TFII expression. In accordance with mouse models, expression of numerous genes important for electron transport chain and mitochondrial ribosome function was repressed in COUP-TFII-overexpressing cells (Fig 4E). Most importantly, gene signatures of “Respiratory_Electron_Transport” and “Organellar_Ribosome” were also downregulated in DA neurons of sporadic PD patients similar to those shown in OE mice (Fig 4F). These results suggest the possibility that elevated COUP-TFII expression in DA neurons causes mitochondrial dysfunction by transcriptional repression of genes critical for mitochondrial function.

Aldehyde dehydrogenases (ALDHs), a family of enzymes that reduce oxidative stress in cells by detoxifying reactive aldehydes, have been shown to be downregulated in brain tissues of PD patients [37]. ALDHs play a critical role in the metabolism of dopamine by converting toxic 3,4-dihydroxyphenylacetaldehyde (DOPAL) to DOPAC [38–40]. Several ALDH genes were repressed in OE mice (Fig 4G). If this is the case, we expect that dopamine metabolite levels would change as well. As shown in S4D Fig, DOPAC levels in OE mice were decreased, which is associated with decreased levels of dopamine. Moreover, the ratio of DOPAC to DOPAL in the striatum was significantly decreased in OE mice (Fig 4H), suggesting that the overall enzymatic activity was reduced. Consistent with decreased ALDH activity, the ratio of DOPAL to DA was significantly increased in OE mice (Fig 4H). Consistently, cell culture experiments indicate that several genes in the gene family of ALDHs were also repressed in COUP-TFII-overexpressing cells (Fig 4I). Since mitochondrial function and enzymes that metabolize the toxic DOPAL are defective, it was expected that ROS levels would be increased. Indeed, we found increased levels of intracellular ROS signals in COUP-TFII-overexpressing cells (Fig 4J). Taken together, these data indicate that elevated COUP-TFII expression impairs mitochondrial function and its ability to relieve oxidative stress in DA neurons, resulting in enhanced vulnerability to neurodegeneration.

### Increased damaged mitochondria and electron-dense vacuoles in OE mice

Next, we performed transmission electron microscopy (TEM) to analyze the neurons in the SN*pc* of Ctrl and OE mice. A significant increase of damaged mitochondria with decreased cristae was observed in the neurons of OE mice as compared to Ctrl mice (S5A Fig). In addition, there were more electron-dense vacuoles in the neurons of OE mice in comparison to Ctrl mice at 10 months of age (S5B Fig; arrow heads), whereas such vacuoles were rarely detected in both Ctrl and OE mice at 3 weeks of age (S5C Fig). Higher-magnification views visualized the ultrastructure of these electron-dense vacuoles, which consisted of autophagosome, autolysosome, mitochondria-like organelles and lipofuscin granules (S5D Fig). The accumulation of electron-dense vacuoles confirms an increasing level of mitochondrial damage in the neurons of OE mice.

### Down-regulation of COUP-TFII expression in DA neurons attenuates the disease progression of MitoPark mice

**Fig 5:**
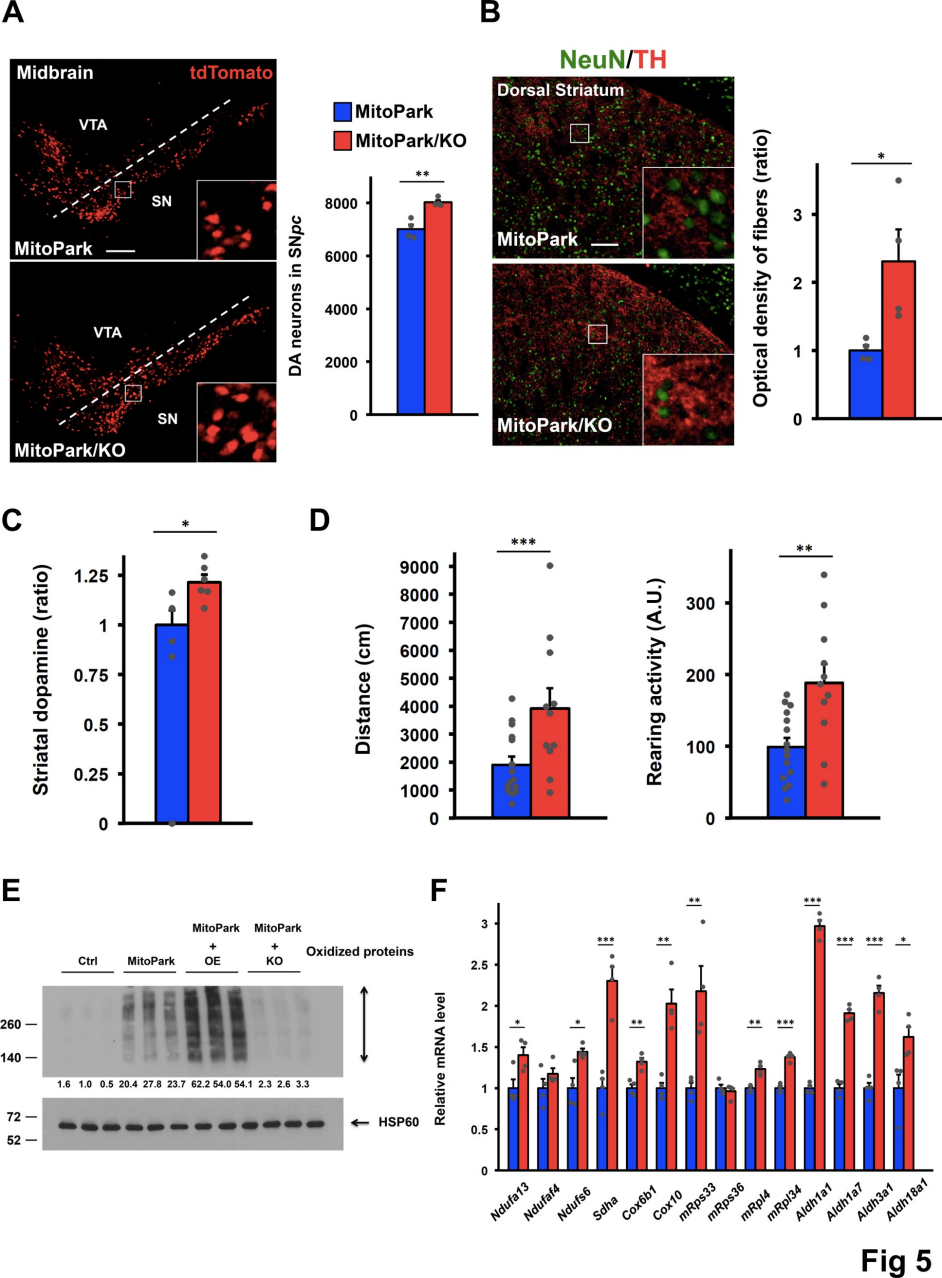
*COUP-TFII* deletion in DA neurons attenuates the disease progression of MitoPark mice. **(A)** Representative images (left) and quantification (right) of DA neurons in the ventral midbrain of 13- to 15-week-old MitoPark and MitoPark/KO mice (higher-power views in the insets). n = 4/group. Scale bar, 200 μm. **(B)** Representative images (left) and quantification (right) of DA axonal projections to the dorsal striatum of 13- to 15-week-old mice after double staining with anti-TH and anti-NeuN antibodies (higher-power views in the insets). n = 4/group. Scale bar, 100 μm. **(C)** Relative total striatal dopamine of 13- to 15-week-old mice. n = 4-6/group. **(D)** Open-field assay recorded for 13- to 15-week-old MitoPark (n = 15) and MitoPark/KO (n = 11) mice. **(E)** Western blot of mitochondrial oxidized proteins from the SN*pc* of 13- to 15-week-old mice. The numbers indicate normalized ratios compared to control. n = 3/group. **(F)** mRNA levels of key genes in the network of mitochondrial pathways from the ventral midbrain of 13- to 15-week-old mice. n = 4/group. **(A-F)** *p < 0.05; **p < 0.01; ***p < 0.001 compared to MitoPark mice (*t*-test). Mean ± SEM.

In order to test whether reduction of COUP-TFII expression can slow down the disease progression of PD, COUP-TFII conditional knockout (KO) mice were bred into the MitoPark background. At 13–15 weeks of age, MitoPark/KO mice displayed a small but significant 15% increase in the number of DA neurons relative to MitoPark mice (Fig 5A). In contrast, DA innervation in the dorsal striatum of MitoPark/KO mice was drastically increased as compared to MitoPark mice (Fig 5B). These results indicate that loss of COUP-TFII expression not only slows down neurodegeneration but also keeps surviving DA neurons healthy. In addition, total striatal dopamine was elevated in MitoPark/KO mice (Fig 5C). Most importantly, motor functions of MitoPark/KO mice were greatly recovered (Fig 5D). To assess the effect of ROS in our mouse models, we measured oxidized mitochondrial proteins from the SN*pc* of mice at 13–15 weeks of age. Oxidized mitochondrial proteins were used as surrogate markers to reflect long-term ROS levels in animals. The results showed that MitoPark/OE mice had significantly more oxidized mitochondrial proteins as compared to MitoPark mice, whereas oxidized mitochondrial proteins were greatly decreased in MitoPark/KO mice (Fig 5E). These results, which are consistent with our previous report [16], clearly support the importance of interplay between COUP-TFII and mitochondrial ROS in our mouse models. Furthermore, mitochondrial gene expression in MitoPark/KO mice was increased compared to MitoPark mice, indicating that COUP-TFII directly or indirectly regulates the network of mitochondrial pathways (Fig 5F). Taken together, our data suggest that loss of COUP-TFII expression is able to maintain more healthy DA neurons and partially attenuate the disease progression of MitoPark mice.

## Discussion

Although among 10% of PD patients, both familial and sporadic cases [5,6] can be linked to multiple genetic changes, the etiology PD is largely undefined. Aside from genetic predisposition, exposure to environmental toxins, such as oxidative stressors and mitochondrial complex I inhibitors, has been strikingly and positively associated with PD [8]. Chief among the defects observed in PD patients is a noted loss of DA neurons, which is accompanied by an increase in oxidized-DNA, -lipids, and -proteins in brain tissues of PD patients [11–13], suggesting that a high level of oxidative stress likely rendered DA neurons susceptible to cell death. Based on studies of limited cases, the mechanisms in the pathogenesis of PD have centered on mitochondrial dysfunction and defective autophagy [9]. However, how the vast majority of sporadic PD patients suffer from neurodegeneration without clear evidence of genetic changes or exposure to environmental toxins remains unknown. A popular theory is the “multi-hit” hypothesis, which suggests that a combination of several risk factors leads to the development of sporadic PD [21]. While GWAS genomic analysis has identified numerous loci associated with PD [19,20], it has yet to “predict” PD so far. Hence, discovering potential contributors or modifiers to PD beyond the genome that are possibly influenced by the environment or genetic predisposition may help understand the etiology and pathogenesis of sporadic PD.

In the present study, we demonstrated that COUP-TFII expression was elevated in PD patients and the mouse model by analyzing public datasets. Although mice with elevated COUP-TFII expression in DA neurons only displayed mild DA neurodegeneration, COUP-TFII overexpression clearly accelerated progressive phenotypes of MitoPark mice, a progressive PD mouse model with impaired respiratory chain function in DA neurons [30]. Our studies suggest that COUP-TFII can augment oxidative stress through transcriptional repression of genes important for mitochondrial electron transport chain and mitochondrial ribosome function, which can in turn enhance COUP-TFII expression in a positive-feedback loop. COUP-TFII plays similar roles on inhibition of mitochondrial function in cases of heart failure [16]. In the heart, elevated COUP-TFII expression is sufficient to induce lethal dilated cardiomyopathy, while in the brain, the loss of DA neurons in mice with elevated COUP-TFII expression is not sufficient to cause motor dysfunction. In the brain, loss of more than 50% of dopamine/DA projections is required before defects in motor functions are elicited, while in the heart, loss of lower numbers of cardiomyocytes may lead to heart failure. Furthermore, unlike in humans, mice seem to be less susceptible to PD. For instance, monogenic mutations in PARKIN account for human PD, while a second hit on mitochondria is necessary to show neurodegeneration in mice [41]. Likewise, enhanced COUP-TFII expression alone in DA neurons did not lead to severe neurodegeneration in mice, but it did accelerate the initiation and progression of phenotypes exhibited by MitoPark mice. Perhaps these reasons may explain why symptoms driven by COUP-TFII-mediated mitochondrial dysfunction in cardiomyocytes are more prominent in comparison to those in neurons.

From the retrospective studies, estimates of the loss of DA neurons in the SN*pc* and markers in the striatum are about 30% and 50–70%, respectively, at the time of motor symptom onset in PD patients [42]. This is consistent with the fact that the loss of striatal DA markers is more profound than that of DA neurons in the SN*pc* from postmortem samples. It is likely that some of DA neurons in the SN*pc* of MitoPark or MitoPark/OE mice are already damaged and fail to project to the striatum. Thus, over estimation of healthy DA neurons may contribute to the observable greater loss of striatum projection than the loss of SN*pc* DA neurons. Our mouse models recapitulate the disease progression of PD, in which a greater decline in axonal projections than actual DA neuron loss is observed in PD patients.

In sporadic PD patients, dysregulated transcriptional profiling of the electron transport chain and partial deficiency of mitochondrial complex I have been documented [9]. The reduced complex I activity can be attributed to the accumulation of damaged mitochondria by exposure to environmental toxins, poor mitochondrial quality control, or misfolded protein aggregation [9]. However, mechanisms underlying the dysregulated transcriptional profiles of the electron transport chain in sporadic PD patients are not well understood. Based on previous reports [43], it is reasonable to speculate the presence of master transcription factors that control neuronal survival or cell death. For instance, Lmx1b is required for the autophagic-lysosomal pathway, maintaining the integrity of DA terminals and DA neuronal survival [44]. It was also reported that Lmx1a/b regulates mitochondrial functions and survival of adult midbrain DA neurons [45,46], whereas continuous up-regulation of the canonical pathway of NF-κB in the SN leads to DA neuron loss [47]. Based on the current and previous studies [16], COUP-TFII is one of the transcription factors that negatively regulate mitochondrial pathways. We believe that elevated COUP-TFII expression in PD patients at least partially (if not fully) contributes to abnormal transcriptional profiles of genes important for the electron transport chain. Given that enhanced COUP-TFII expression led to a repression of mitochondrial ribosome genes, and that several mitochondrial ribosome genes are mapped to loci associated with mitochondrial diseases arising from impaired oxidative phosphorylation [48], our studies suggest that COUP-TFII contributes to mitochondrial dysfunction in PD. In addition, expression of the cytosolic detoxifier ALDH genes was unexpectedly repressed in a DA cell line as well as in DA neurons of mice overexpressing COUP-TFII. These enzymes are known to relieve oxidative stress from the mitochondria of DA neurons or to control dopamine metabolism by reducing the DOPAL to DA ratio [37] in DA neurons. Indeed, we observed increased DOPAL to DA ratios in mice with elevated COUP-TFII expression, suggesting that reduced enzymatic activity failed to effectively remove the toxic metabolite, DOPAL, which is detrimental to DA neurons. In postmortem brain tissues of PD patients, decreased levels of ALDHs and increased DOPAL to DA ratios have been reported [37,40], indicating the importance of ALDHs in the pathogenesis of PD. In addition, animal studies provided strong evidence for ALDH-mediated neuroprotection and DOPAL-induced neurodegeneration [49,50]. Hence, our mouse models lend support to both the “mitochondrial dysfunction” and “catechol-aldehyde” hypotheses, suggesting that oxidative stress triggers PD.

Ultrastructural examination of neurons in the SN*pc* revealed more damaged mitochondria in mice with elevated COUP-TFII expression as compared to control. This result strengthens our hypothesis on COUP-TFII-mediated mitochondrial dysfunction in DA neurons, analogous to our previous report regarding heart failure [16]. In addition, more electron-dense vacuoles appeared in the neurons of mice with elevated COUP-TFII expression. The presence of autophagic vacuoles and lipofuscin granules is commonly found in postmortem brain tissues of PD patients [51,52]. It is generally believed that these phenotypes arrived from dysregulated networks of mitochondrial pathways and autophagic degradation systems in PD patients. Based on our study, the increase in damaged mitochondria caused by COUP-TFII-mediated mitochondrial dysfunction likely leads to the accumulation of autophagic vacuoles. Although there was no obvious correlation between COUP-TFII-regulated genes and gene signatures of autophagy pathways, the possibility that COUP-TFII may affect autophagy cannot be excluded. Moreover, lipofuscin is a complex mixture with highly oxidized cross-linked non-degradable macromolecules and is considered as an aging marker and a risk factor for neurodegenerative diseases [53]. Accumulation of defective mitochondria derived from aging or neurodegenerative diseases may likely promote lipofuscinogenesis. Regardless of whether lipofuscin actively perturbs cellular metabolism or represents a protective neuronal mechanism, the results obtained from TEM analysis support the notion that elevated COUP-TFII expression in DA neurons generally increases oxidative stress in these cells.

In summary, the present study suggests that mice with elevated COUP-TFII expression specifically in DA neurons exhibited DA neuron loss and acceleration of the disease progression of the PD mouse model, MitoPark, through transcriptional repression of genes essential for mitochondrial function and genes critical for cellular detoxification, ALDHs. Mitochondrial dysfunction together with the lowering of ROS scavenger capacity combine to trigger the enhancement of oxidative stress in DA neurons. Our results suggest that COUP-TFII serves as a secondary hit, rendering the organisms more susceptible to PD. COUP-TFII is an orphan nuclear receptor, which belongs to a family of receptors that contain a ligand binding pocket and can serve as a drug target. Since up-regulation of COUP-TFII in PD patients accelerates the progression of PD phenotypes, down-regulation of COUP-TFII may prolong as well as improve the quality of life of PD patients. Thus, COUP-TFII inhibitors that are effective in repressing its activity could be beneficial for future treatment of PD patients.

## Materials and methods

### Animals

Mice with elevated COUP-TFII expression that carry the *ROSA26*^*CAG-S-COUP-TFII*^ allele and COUP-TFII conditional KO mice that carry the *COUP-TFII flox* allele were used as described [15]. The DA neurons-specific Cre-expressing mice (B6.SJL-*Slc6a3*^*tm1*.*1(cre)Bkmn*^/J), the *Tfam* floxed mice (B6.Cg-*Tfam*^*tm1*.*1Ncdl*^/J), and the *ROSA26*-tdTomato reporter mice (B6.Cg-*Gt(ROSA)26Sor*^*tm9(CAG-tdTomato)Hze*^/J) were acquired from the Jackson Laboratory. Ctrl, OE, MitoPark, and MitoPark/OE littermates were obtained by crossing *Slc6a3*^*IREScre/IREScre*^; *Tfam*^*fl/+*^*; ROSA26*^*CAG-S-COUP-TFII/+*^ males and *Tfam*^*fl/fl*^; *ROSA26*^*tdTomato/tdTomato*^ females. For the rescue experiment, MitoPark and MitoPark/KO mice were generated by crossing *Slc6a3*^*IREScre/IREScre*^; *Tfam*^*fl/+*^; *COUP-TFII*^*fl/+*^ males and *Tfam*^*fl/fl*^; *COUP-TFII*^*fl/fl*^; *ROSA26*^*tdTomato/tdTomato*^ females. All animals used in this study included both males and females. We did not observe any significant phenotypic differences between males and females. All animal experiments strictly adhered to the Guidelines of BCM Animal Care and Use Policies and Procedures approved by the Institutional Animal Care and Use Committee of Baylor College of Medicine and were conducted within the scope of approved animal protocols (AN-113 and AN-1002).

### Analysis of publicly available array data

The samples in selected groups from GEO DataSets were all included. The following probes for COUP-TFII were analyzed, 209121_x_at (GSE7621), 209119_x_at (GSE20141), 1444229_at (GSE4758), ILMN_2094360 (GSE46798), 1336 (GSE4788), and 1416160_at (GSE17542). For GSE46798, “Corr” and “A53T” groups were used. For GSE4788, “Substantia Nigra Control RMA, MAS 5, dChip” and “Substantia Nigra MME RMA, MAS 5, dChip” groups were used. For GSE17542, “Control SN” and “2 day MPTP treated SN” groups were used.

### Cell lines

SH-SY5Y (CRL-2266, female-derived, ATCC) cells were cultured in DMEM/F-12 (Gibco) supplemented with fetal bovine serum (FBS) (10%, Gibco), penicillin/streptomycin (1:100, P7539, Sigma), and L-glutamine (1:100, P7513, Sigma). DA-like SH-SY5Y neuroblastoma cells were obtained from culturing in the differentiation media consisting of DMEM/F-12 supplemented with FBS (1%) and retinoic acid (20 μM) for 7 days. After differentiation, H_2_O_2_ (H1009, Sigma), MPP^+^ (D048, Sigma), rotenone (R8875, Sigma), or paraquat (#36541, Sigma) was added in the culture media for 24 hours. Alternatively, DA-like SH-SY5Y neuroblastoma cells were preincubated with NAC (Enzo Life Sciences) for an hour and further incubated with MPP^+^ for 24 hours. For the study of epigenetics, DA-like SH-SY5Y neuroblastoma cells were treated with 5-aza-2'-deoxycytidine (A3656, Sigma) and/or Trichostatin A (T1952, Sigma). To demonstrate the effect of SNCA on COUP-TFII expression, stable SH-SY5Y cell lines overexpressing SNCA-WT or SNCA-A53T were generated by transduction of the pInducer20-*FLAG*-*SNCA-WT/A53T* lentiviral vectors (pInducer20 vector and plasmids carrying SNCA-WT or SNCA-A53T cDNA purchased from Addgene) and subsequently selected for G418 resistance (#11811031, 1 mg/mL, Thermo Scientific). SNCA-WT or SNCA-A53T was induced by doxycycline (250 ng/mL, Clontech) for 3 days with or without NAC treatment. For mechanistic studies, the stable N27 cell line (SCC048, female-derived, EMD Millipore) overexpressing COUP-TFII under the control of doxycycline was established by transduction of the pInducer20-*FLAG*-*COUP-TFII* lentiviral vector and subsequently selected for G418 resistance (500 μg/mL). This rat DA cell line was maintained in DMEM supplemented with tetracycline-free FBS (10%, Clontech), penicillin/streptomycin, and L-glutamine. The differentiation protocol required culturing in the media supplemented with tetracycline-free FBS (2%) and dibutyryl cAMP sodium salt (2mM, D0260, Sigma) for 4 days. Doxycycline (250 ng/mL) was added to induce COUP-TFII expression 2 days prior to experimentation. Rabbit anti-COUP-TFII (1:1000, #6434, Cell Signaling), rabbit anti-SNCA (1:1000, #2642, Cell Signaling), and rabbit GAPDH (1:1000, sc-25778 HRP, Santa Cruz) were used for protein analysis.

### Reverse transcription quantitative PCR (RT-qPCR)

Total RNA was extracted using the TRIzol (LS) reagent (#15596026 or #10296010, Invitrogen) according to the protocol provided by the manufacturer. Reverse transcription reactions were carried out using the First Strand cDNA Synthesis Kit for RT-PCR (#11483188001, Sigma) to make cDNA according to the manufacturer’s guide. qPCR analysis was performed on the StepOnePlus Real-Time PCR System (Applied Biosystems) using the FastStart Universal SYBR Green Master (#4913850001, Sigma). Each cycle of the qPCR consisted of 15 seconds at 95°C for denaturing and 60 seconds at 60°C for annealing and extension with a total of 40 cycles performed. Relative mtDNA copy number in the N27 rat DA neural cell line was also determined by qPCR. Primer sequences are shown in S2 Table. DNA methylation and chromatin immunoprecipitation (ChIP)

Methylated DNA fragments were isolated by the CpG island-specific Promoter Methylation PCR Kit (MP1100, Affymetrix). ChIP assay was conducted following the ChIP-IT Express Enzymatic Kit (#53009, Active Motif) with rabbit anti-histone H3ac (pan-acetyl, #61637, Active Motif) antibody and normal rabbit IgG (#2729, Cell Signaling). Recovered DNA fragments from both assays were subjected to qPCR described above. Primer sequences are shown in S2 Table.

### Isolation of DA neurons

Isolation of adult mouse neurons from fresh ventral midbrain was performed according to a protocol published in Nature Protocols [54]. During the procedures, the working solutions were supplemented with trehalose (5%, T0167, Sigma) to enhance cell viability [55]. The digestion medium was further added with DNase I (100 units/mL, LK003172, Worthington). The suspended cells from “release of cells from tissue” step were added with an RNase inhibitor (1:100, N2615, Promega), SYTOX Green Dead Cell Stain (1:1000, S34860, Invitrogen), and NucBlue Live ReadyProbes Reagent (2 drops/mL, R37605, Molecular Probes) to facilitate cell sorting of tdTomato^+^ live DA neurons on a FACSAria II flow cytometer (BD Biosciences).

### Tissue preparation and immunohistochemistry

Anesthetized mice were transcardially perfused with ice-cold phosphate buffered saline (PBS) (Gibco) and subsequently with ice-cold paraformaldehyde in PBS (4%, #441244, Sigma). Mouse brains were then isolated, post-fixed overnight at 4°C, and processed for 7-μm-thick paraffin-embedded or 30-μm-thick frozen tissue sections. In preparation of frozen tissues, mouse brains were cyroprotected by sucrose in PBS (15% and 30%, S0389, Sigma) before being embedded in O.C.T. compound (Tissue-Tek). Immunohistochemistry was performed as described previously [15]. Primary antibodies included rabbit anti-tyrosine hydroxylase (TH) (1:1000, #657012, Millipore), mouse anti-TH (1:400, MAB318, Millipore), rabbit anti-tdTomato (1:200, #600-401-379, Rockland), mouse anti-tdTomato (1:200, TA180009, Origene), rat anti-DAT (MAB369, 1:400, Millipore), rabbit anti-aromatic L-amino acid decarboxylase (AADC) (1:400, ab3905, Abcam), rabbit anti-MYC-Tag (1:100, #2278, Cell Signaling), and rabbit anti-NeuN (1:200, #12943, Cell Signaling). Secondary antibodies included biotin-SP-conjugated F(ab’)_2_ donkey anti-rabbit IgG (1:200, #711-066-152, Jackson ImmunoResearch), Alexa Fluor 594 donkey anti-mouse IgG (1:500, A21203, Invitrogen), Alexa Fluor 594 donkey anti-rabbit IgG (1:500, A21207, Invitrogen), Alexa Fluor 488 donkey anti-mouse IgG (1:500, A21202, Invitrogen), Alexa Fluor 488 donkey anti-rabbit IgG (1:500, A21206, Invitrogen), and Alexa Fluor 488 donkey anti-rat IgG (1:500, A21208, Invitrogen). DAPI (1:1000, #62248, Thermo Scientific) was used for nuclear staining. For chromogenic staining, signals were amplified by Vectastain ABC Elite (PK-6100, Vector Laboratories) followed by ImmPACT DAB (SK-4105, Vector Laboratories); for immunofluorescent staining, Tyramide SuperBoost Kits (B40932 and B40935, Thermo Scientific) were used, if needed.

### Laser capture microdissection

Analysis of DNA methylation in CpG islands in the COUP-TFII gene locus of MitoPark mice was performed with ArcturusXT Laser Capture Microdissection System (Thermo Scientific) following the manufacturer’s instructions. Briefly, DA neurons in the SN*pc* from 7-μm-thick paraffin-embedded tissue sections were identified by immunostaining with anti-TH antibodies described above. DA neurons from an average of twenty sections were collected by laser capture microdissection. DNA was extracted and purified using the QIAamp DNA FFPE Tissue Kit (#56404, Qiagen). Methylated DNA was fragmented by the treatment of the restriction enzyme DpnII (NEB) and then isolated by the EpiXplore™ Methylated DNA Enrichment Kit (#631962, Takara). Recovered DNA fragments were subjected to qPCR described above. Primer sequences are shown in S2 Table.

### Unbiased stereology

Unbiased stereology, using the optical fractionation method in the software Stereo Investigator (MicroBrightField), was used to estimate DA neurons and total neurons in the SN*pc* or the VTA. For this experiment, every 4^th^ 30-μm-thick frozen tissue sections covering the entire region of the SN*pc* and the VTA were used, and neurons from the same hemisphere were counted using the 40X lens. DA neurons were visualized by strong endogenous fluorescence from tdTomato (without the use of antibodies), and total neurons were counterstained by NeuroTrace green fluorescent Nissl stain (N21480, Molecular Probes) (S6 Fig). A fixed counting frame of 50 μm x 50 μm and a sampling grid size of 150 μm x 150 μm were set. A coefficient of error of < 0.15 was accepted.

### Quantification of striatal DA innervation

Striatal DA innervation was carried out by immunostaining with DA markers (TH, DAT, and AADC). Quantification of DA innervation was then conducted by means of optical densitometry using ImageJ (NIH). The values of pixel brightness measured from 7-μm-thick paraffin-embedded tissue sections were corrected for the nonspecific background by subtracting values obtained from nearby brain regions outside the striatum. To assess DA axonal projections from the SN*pc*, optical densitometry was measured from the dorsal sector of the striatum. For those from the VTA, the ventral sector of the striatum was used. An average of sixty sections were prepared for each mouse. The comparable multiple sections in all the mice were used for staining and quantitation. The data were presented as a ratio compared to control mice.

### Dopamine and metabolite measurements

Striata were quickly dissected and frozen from euthanized mice. Dopamine, metabolites, and other neurotransmitters including serotonin, norepinephrine, and epinephrine were measured by the Neurochemistry Core at the Vanderbilt University using high-performance liquid chromatography (HPLC) with electrochemical detection (ECD). The data were normalized to total protein and presented as a ratio compared to control mice. We also measured striatal catechols including DOPA, DA, DOPAC, and DOPAL by HPLC-ECD as described previously [40].

### Behavioral tests

For the open-field assay, mice were habituated in the testing room for 30 minutes and then placed in the center of an open-field space. Horizontal and vertical activities were monitored by the VersaMax system (VersaMax Legacy Open Field, Omnitech Electronics) for a period of 30-minutes. The data were pooled into fifteen 2-minute intervals and analyzed. In order to examine the effects of L-DOPA, mice were intraperitoneally injected with methyl L-DOPA hydrochloride (20 mg/kg, D1507, Sigma) dissolved in normal saline (Henry Schein) containing benserazide hydrochloride (5 mg/kg, B7283, Sigma). The test was carried out for a period of 4-hours. For the rotarod performance test, mice were placed on an accelerating rotarod apparatus (#47650, UGO Basile), which accelerated from 4 to 40 rpm over a 5-minute period, and given 4 trials on each of 2 consecutive days with 30-minute rest intervals. The amount of time for each mouse to fall from the rod was recorded. The first day was considered as the training day. The data from the 4 trials on the second day were analyzed and presented. For the pole test, mice were placed onto the top end of a pole covered with gauze pads, and the time they remained on the pole was recorded. The maximum time for this test was 60 seconds.

### Gene expression analysis

The ventral midbrain tissues were dissected and snap-frozen in liquid nitrogen. The samples were sent to Q^2^ Solutions (Morrisville, North Carolina, USA) for RNA extraction, sequencing, and analysis. Briefly, high-quality RNA samples were converted into cDNA libraries using the Illumina TruSeq Stranded mRNA sample preparation kit (RS-122-2103, Illumina) and then sequenced through HiSeq-Sequencing-2x50bp-PE sequencing on an Illumina sequencing platform. After sequencing, the analysis was performed using the in-house developed RNAv9 pipeline from the company. Genes with fold change of more than log_2_ 0.2 were uploaded and analyzed by the DAVID Functional Annotation Tool. GSEA was performed using the GSEA v3.0 software. The GSEAPreranked tool was run with gene sets derived from GO and Reactome pathway databases. The heatmap was generated by the GenePattern module. The RNAseq data have been deposited in the GEO database under accession code GSE132874.

### Mitochondrial respiration assay

XF24 extracellular flux analyzer (Seahorse Bioscience) was used to measure the oxygen consumption rate following the manufacturer’s instructions. Briefly, an XF24 cartridge was equilibrated with the XF calibrant overnight at 37°C. DMEM was used to prepare reagents and serve as the assay buffer. The compounds used for mitochondrial respiration assay were oligomycin (0.5 μM, O4876, Sigma), carbonyl cyanide-4-phenylhydrazone (FCCP) (2.5 μM, C2920, Sigma), and rotenone (0.5 μM). COUP-TFII-overexpressing cells were used in this experiment. Data were pooled from repeated independent experiments (5 replicates/each).

### Flow cytometry

To measure mitochondrial membrane potential and ROS in live cells, the cells were added separately with tetramethylrhodamine (TMRM) (100 nM, T668, Invitrogen) and the CellROX Orange reagent (5 μM, C10443, Molecular Probes) into the media and incubated for 30 minutes at 37°C. Then the media were removed, and the cells were washed with PBS 3 times prior to analysis. The cells in ice-cold PBS were analyzed immediately on an LSRFortessa flow cytometer (BD Biosciences).

### Transmission electron microscopy

Anesthetized mice were transcardially perfused with normal saline and subsequently treated with modified Karnovsky’s fixative (2% paraformaldehyde and 2.5% glutaraldehyde, Electron Microscopy Sciences) in a sodium cacodylate buffer (0.1 M, pH 7.4, 320 mOsm, Electron Microscopy Sciences). The SN*pc* and the dorsal striatum were dissected and sliced into 1mm-thick slices. Tissues were placed into a scintillator vial with the buffered fixative and placed on a rotator for 3 days at 4°C. Then the tissues were processed inside a BioWave microwave processing system (Ted Pella). Briefly, the samples were fixed again, followed by sodium cacodylate buffer rinses, post-fixed with osmium tetroxide (1%, Electron Microscopy Sciences) in a sodium cacodylate buffer, and rinsed with water. Ethanol concentrations (25–100%, Decon Laboratories) were used for the initial dehydration series, followed with propylene oxide (Electron Microscopy Sciences) as the final dehydrant. The samples were gradually infiltrated with 4-step changes of propylene oxide and resin (Electron Microscopy Sciences) ratios into pure resin. The samples were allowed to stay in pure resin overnight on a rotator. The samples were then embedded into regular capsules (Ted Pella) and cured in the oven for 5 days at 62°C. The polymerized samples were sectioned at 50 nm. The grids were then stained with uranyl acetate (1%, Electron Microscopy Sciences) for 10 minutes, followed by 2.5% lead citrate (2.5%, Electron Microscopy Sciences) for 2 minutes one day before TEM examination. The grids were viewed in a transmission electron microscope at 80kV (JEM-1400 plus, Jeol). The images were captured using a digital camera (XR16, Advanced Microscopy Techniques). To assess damaged mitochondria in the SN*pc*, every single mitochondrion in the neurons was analyzed. Those with poorly defined cristae were counted as damaged mitochondria.

### Oxidized mitochondrial proteins

The protocol for isolation of mitochondria was used according to the reference [56]. The measurement of oxidized mitochondrial proteins was carried out on freshly isolated mitochondria form the SN*pc* by the OxyBlot Protein Oxidation Detection Kit (S7150, Millipore). The rabbit anti-HSP60 antibody (1:1000, #12165, Cell Signaling) was used for the loading control.

### Statistical tests

Two-tailed *t*-test or one-way ANOVA Fisher’s LSD post hoc test was used in this study unless stated otherwise.

## Supporting information

S1 Fig COUP-TFII expression is upregulated in animal and cell models of PD, and elevated COUP-TFII expression in DA neurons leads to mild degeneration in mice, related to Fig 1. **(A)** COUP-TFII mRNA levels in DA neurons derived from human iPS cells of PD patients carrying the SNCA-A53T mutation from GSE46798 (n = 3/group). SNCA-WT, the A53T mutation corrected to wild-type prior to differentiation. **(B)** COUP-TFII mRNA levels in the SN tissues (left) of control and neurotoxin prodrug MPTP-treated mice from GSE4788 (n = 4/group) and in DA neurons (right) of the SN tissues from control and MPTP-treated mice from GSE17542 (n = 3/group). **(C)** COUP-TFII mRNA levels in the differentiated SH-SY5Y cell line after 24-hour treatment with neurotoxin MPP^+^, broad-spectrum pesticide rotenone, or herbicide paraquat. n = 3/group. One-way ANOVA Fisher’s LSD post hoc test. **(D)** COUP-TFII mRNA levels in the SY-SH5Y cells overexpressing SNCA-WT or SNCA-A53T after 3-day induction in the absence or presence of 2 mM N-acetyl-L-cytsteine (NAC) antioxidant. n = 5 replicates/group. Two-way ANOVA Fisher’s LSD post hoc. This experiment was independently repeated and produced with similar results. **(E)** CpG islands and histone H3K27 acetylation at the gene locus of COUP-TFII from the UCSC Genome Browser as a reference for epigenetic studies. TSS, transcription start site; ATG, translation start site. **(F)** Representative images (left) of DA neurons in the ventral midbrain of 16- to 17-week-old control (Ctrl) and COUP-TFII overexpression mice (OE) after co-staining with anti-tyrosine hydroxylase (TH) and anti-MYC-Tag antibodies (higher-power views in the insets) and COUP-TFII mRNA levels (right) in isolated DA neurons of the ventral midbrain from 9-week-old mice (n = 4/group). Scale bar, 200 μm. **(G)** Representative images (left) and quantification (right) of DA axonal projections to the dorsal striatum of 16- to 17-week-old mice after staining with anti-aromatic L-amino acid decarbosylase (AADC) antibodies (higher-power views in the insets). n = 5/group. Scale bar, 100 μm. **(H)** Relative total striatal dopamine of 1.5-year-old mice. n = 3/group. **(I)** Pole test recorded for 1.5-year-old mice. n = 3/group. **(A-I)** *p < 0.05; **p < 0.01; ***p < 0.001 compared to control (*t*-test if not indicated). Mean ± SEM.
(TIF)

S2 Fig Elevated COUP-TFII expression in MitoPark mice results in shorter survival time and accelerates the loss of axonal degeneration in the dorsal striatum, however, with minimal effects in the ventral striatum, related to Fig 2. **(A)** Relative recovery ratio of methylated DNA fragments from the gene locus of COUP-TFII in the SNpc of Ctrl and MitoPark mice at 9–10 weeks of age. n = 5/group. **(B)** COUP-TFII expression in the SN*pc* of Ctrl and MitoPark mice at 9–10 weeks of age. The numbers indicate normalized ratios compared to control. n = 3/group. **(C)** Body weights of Ctrl, OE, MitoPark, and MitoPark/OE mice at 16 and 24 weeks of age. n = 5/group. **(D)** Survival rate over time. n = 9/group. The Kaplan-Meier log rank test was performed to compare MitoPark and MitoPark/OE mice. **(E)** Representative images of DA axonal projections to the dorsal striatum of MitoPark and MitoPark/OE mice at 6, 9, and 16 weeks of age after double staining with anti-TH and anti-NeuN antibodies. Scale bar, 100 μm. **(F)** Representative images (left; marked regions) and quantification (right) of DA neurons in the ventral tegmental area (VTA) of 16- to 17-week-old mice after staining with anti-tyrosine hydroxylase (TH). n = 4/group. Scale bar, 200 μm. **(G)** Representative images (left) of DA axonal projections to the striatum after staining with anti-TH antibodies and quantification (right) of TH^+^ fibers in the ventral striatum of 16- to 17-week-old mice. D, dorsal; V, ventral. n = 5/group. Scale bar, 1 mm. **(H)** Total striatal serotonin (5-HT) in 9- to 10-week-old mice. n = 5/group. **(A-H)** *p < 0.05; **p < 0.01; ***p < 0.001. Mean ± SEM. One-way ANOVA Fisher’s LSD post hoc test unless stated otherwise.
(TIF)

S3 Fig MitoPark mice at 23–24 weeks of age start to show responsiveness to L-DOPA treatment, related to Fig 3. **(A)** Open-field assay recorded for 15- to 16-week-old Ctrl, OE, MitoPark, and MitoPark/OE mice treated with saline. n = 6-7/group. **(B)** Open-field assay recorded for 23- to 24-week-old mice treated with L-DOPA (20 mg/kg) (upper) or saline (lower). n = 6/group. **(A-B)** The arrows indicate reagent addition steps. Before, averaged activity from 0 to 30 minutes (pre-Saline/L-DOPA treatment); After, averaged activity from 75 to 105 minutes (post-Saline/L-DOPA treatment). *p < 0.05; **p < 0.01; ***p < 0.001. Mean ± SEM. One-way ANOVA Fisher’s LSD post hoc test.
(TIF)

S4 Fig Elevated COUP-TFII expression dysregulates mitochondrial pathways and dopamine metabolites, related to Fig 4. **(A)** Gene Ontology analysis of the transcriptional profiles from 2-month-old Ctrl and OE mice in the Kyoto Encyclopedia of Genes and Genomes. **(B)** Mitochondria oxygen consumption rates in the N27 rat DA neural cell line. n = 10/group. **(C)** Relative mitochondrial DNA (mtDNA) copy numbers in the N27 rat DA neural cell line. n = 4/group. **(D)** Relative total striatal catechols of 2-month-old Ctrl and OE mice. n = 8/group. **(A-D)** **p < 0.01; ***p < 0.001 compared to control (*t*-test). Mean ± SEM.
(TIF)

S5 Fig Increased electron-dense vacuoles in the SN*pc* of mice with elevated COUP-TFII expression. **(A)** Representative TEM images (left) and quantification (right) of damaged mitochondria (asterisks) in the SN*pc* of 10-month-old Ctrl and OE mice. n = 224 (Ctrl) and 212 (OE) mitochondria from 2 mice. Scale bar, 900 nm. **(B)** Representative TEM images (left) and quantification (right) of electron-dense vacuoles (arrowheads) in the SN*pc* of 10-month-old Ctrl and OE mice. n = 35 (Ctrl) and 34 (OE) neurons from 2 mice. Scale bar, 2 μm. **(C)** Representative TEM images of neurons in the SN*pc* of 3-week-old Ctrl and OE mice. Scale bar, 2 μm. **(D)** Higher-magnification views of various electron-dense vacuoles containing autophagosomes (arrows), autolysosome (arrowheads), mitochondria-like organelles (crosses) and lipofuscin granules (asterisks) in the SN*pc* of OE mice. Scale bar, 300 nm. **(A-D)** N, nucleus; Nc, nucleolus; M, mitochondria. *p < 0.05; ***p < 0.001 compared to control (*t*-test). Mean ± SEM.
(TIF)

S6 Fig Nissl^+^ total neuron counting. Numbers of total neurons were estimated by counting Nissl^+^ neurons in the SN*pc* or VTA, **(A)** refered to Fig 1H, **(B)** refered to Fig 2A, **(C)** refered to S2F Fig, and **(D)** refered to Fig 5A. *p < 0.05; **p < 0.01; ***p < 0.001. Mean ± SEM. *t*-test or One-way ANOVA Fisher’s LSD post hoc test.
(TIF)

S1 Table Fold change of RNAseq, related to Fig 4. (XLSX)

S2 Table Primer sequence, related to Figs 1, 4 and 5. (PDF)
