# Supplementary figures and images for: Elevated COUP-TFII expression in dopaminergic neurons accelerates the progression of Parkinson’s disease through mitochondrial dysfunction
Source: PLoS Genet. 2020 Jun 24;16(6):e1008868. doi: 10.1371/journal.pgen.1008868 (PMC7340320; doi:10.1371/journal.pgen.1008868)

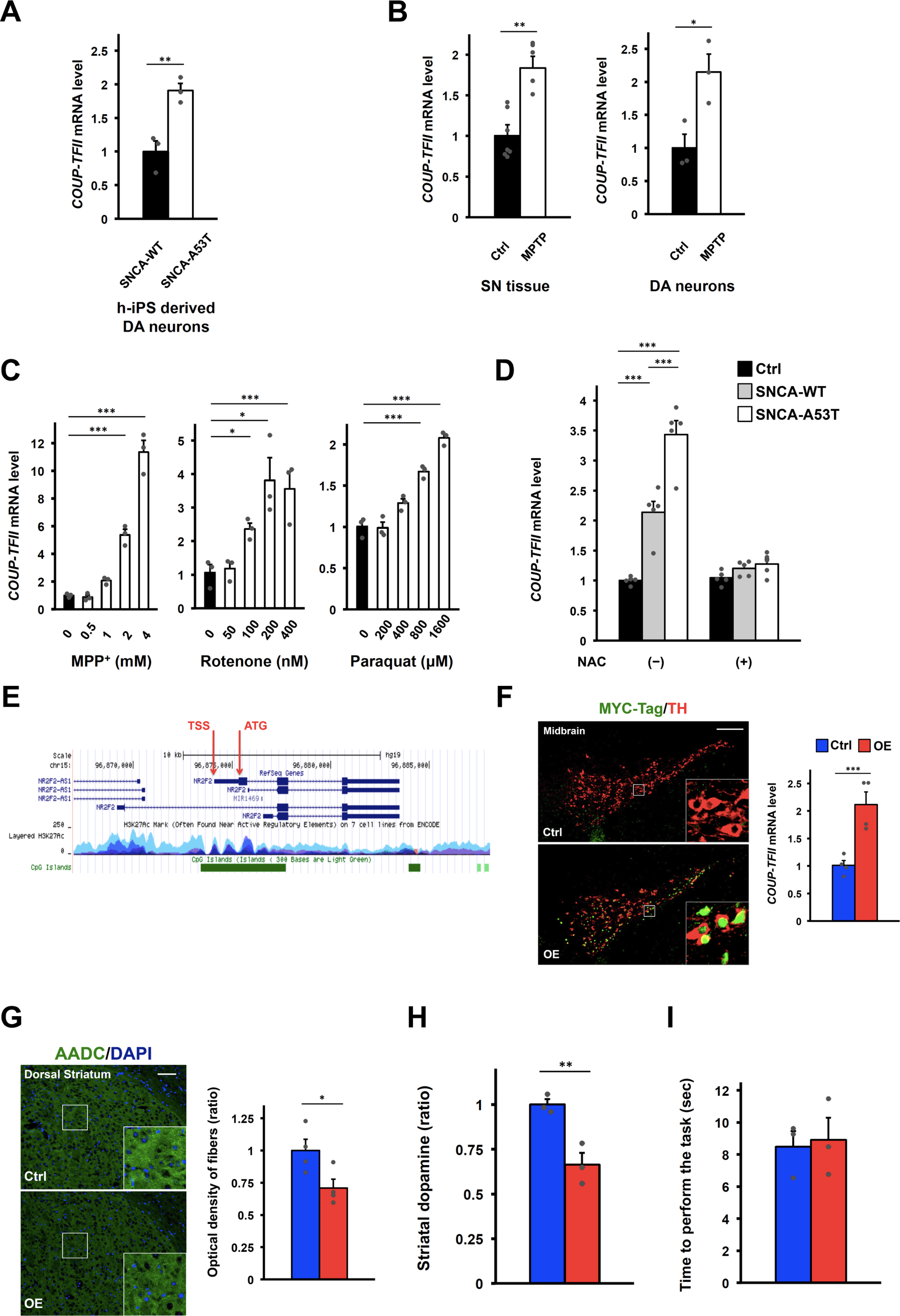

Supplement: S1 Fig — (A) COUP-TFII mRNA levels in DA neurons derived from human iPS cells of PD patients carrying the SNCA-A53T mutation from GSE46798 (n = 3/group). SNCA-WT, the A53T mutation corrected to wild-type prior to differentiation. (B) COUP-TFII mRNA levels in the SN tissues (left) of control and neurotoxin prodrug MPTP-treated mice from GSE4788 (n = 4/group) and in DA neurons (right) of the SN tissues from control and MPTP-treated mice from GSE17542 (n = 3/group). (C) COUP-TFII mRNA levels in the differentiated SH-SY5Y cell line after 24-hour treatment with neurotoxin MPP+, broad-spectrum pesticide rotenone, or herbicide paraquat. n = 3/group. One-way ANOVA Fisher’s LSD post hoc test. (D) COUP-TFII mRNA levels in the SY-SH5Y cells overexpressing SNCA-WT or SNCA-A53T after 3-day induction in the absence or presence of 2 mM N-acetyl-L-cytsteine (NAC) antioxidant. n = 5 replicates/group. Two-way ANOVA Fisher’s LSD post hoc. This experiment was independently repeated and produced with similar results. (E) CpG islands and histone H3K27 acetylation at the gene locus of COUP-TFII from the UCSC Genome Browser as a reference for epigenetic studies. TSS, transcription start site; ATG, translation start site. (F) Representative images (left) of DA neurons in the ventral midbrain of 16- to 17-week-old control (Ctrl) and COUP-TFII overexpression mice (OE) after co-staining with anti-tyrosine hydroxylase (TH) and anti-MYC-Tag antibodies (higher-power views in the insets) and COUP-TFII mRNA levels (right) in isolated DA neurons of the ventral midbrain from 9-week-old mice (n = 4/group). Scale bar, 200 μm. (G) Representative images (left) and quantification (right) of DA axonal projections to the dorsal striatum of 16- to 17-week-old mice after staining with anti-aromatic L-amino acid decarbosylase (AADC) antibodies (higher-power views in the insets). n = 5/group. Scale bar, 100 μm. (H) Relative total striatal dopamine of 1.5-year-old mice. n = 3/group. (I) Pole test recorded for 1.5-year-ol [file pgen.1008868.s001.tif]

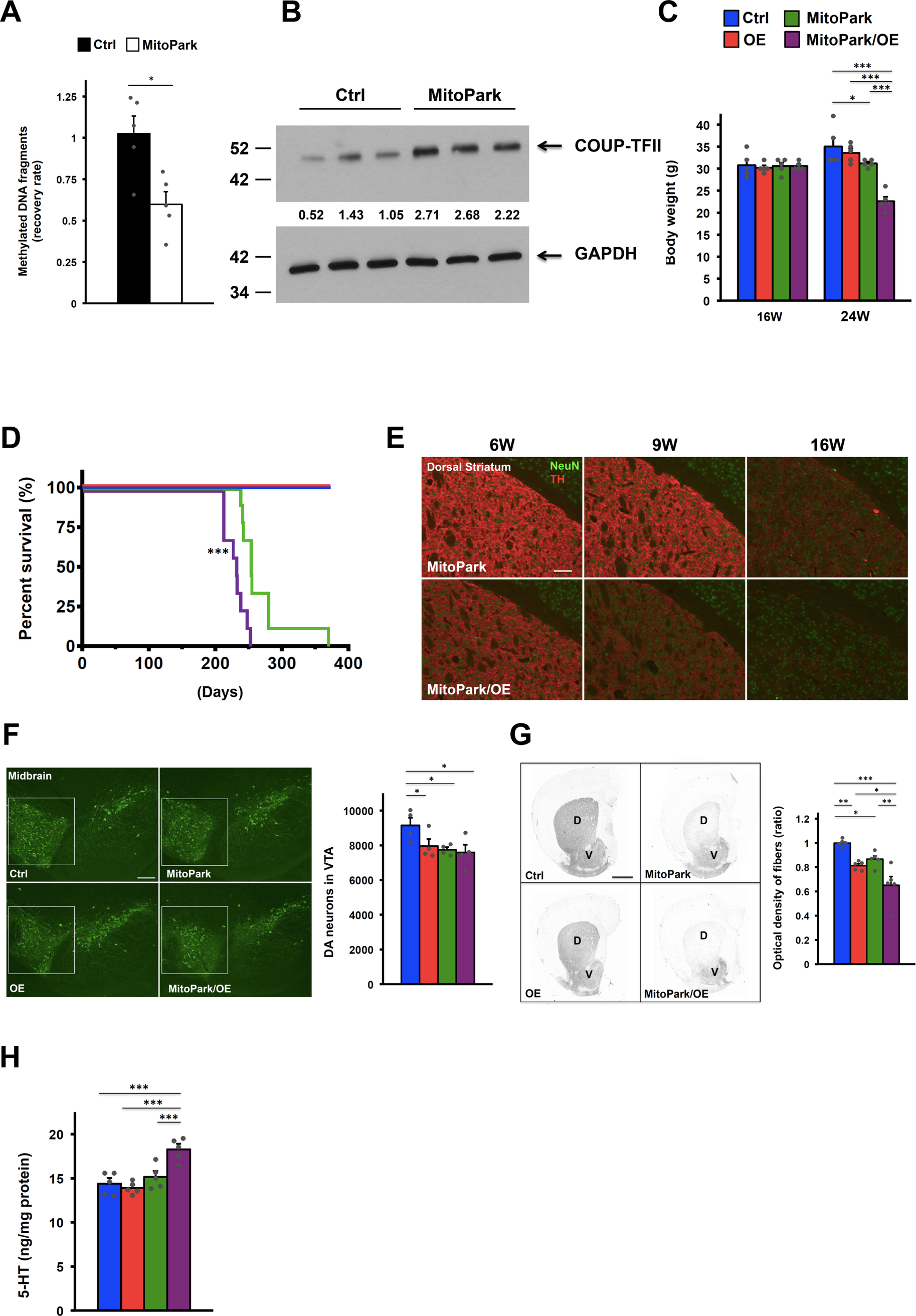

Supplement: S2 Fig — (A) Relative recovery ratio of methylated DNA fragments from the gene locus of COUP-TFII in the SNpc of Ctrl and MitoPark mice at 9–10 weeks of age. n = 5/group. (B) COUP-TFII expression in the SNpc of Ctrl and MitoPark mice at 9–10 weeks of age. The numbers indicate normalized ratios compared to control. n = 3/group. (C) Body weights of Ctrl, OE, MitoPark, and MitoPark/OE mice at 16 and 24 weeks of age. n = 5/group. (D) Survival rate over time. n = 9/group. The Kaplan-Meier log rank test was performed to compare MitoPark and MitoPark/OE mice. (E) Representative images of DA axonal projections to the dorsal striatum of MitoPark and MitoPark/OE mice at 6, 9, and 16 weeks of age after double staining with anti-TH and anti-NeuN antibodies. Scale bar, 100 μm. (F) Representative images (left; marked regions) and quantification (right) of DA neurons in the ventral tegmental area (VTA) of 16- to 17-week-old mice after staining with anti-tyrosine hydroxylase (TH). n = 4/group. Scale bar, 200 μm. (G) Representative images (left) of DA axonal projections to the striatum after staining with anti-TH antibodies and quantification (right) of TH+ fibers in the ventral striatum of 16- to 17-week-old mice. D, dorsal; V, ventral. n = 5/group. Scale bar, 1 mm. (H) Total striatal serotonin (5-HT) in 9- to 10-week-old mice. n = 5/group. (A-H) *p < 0.05; **p < 0.01; ***p < 0.001. Mean ± SEM. One-way ANOVA Fisher’s LSD post hoc test unless stated otherwise. (TIF) [file pgen.1008868.s002.tif]

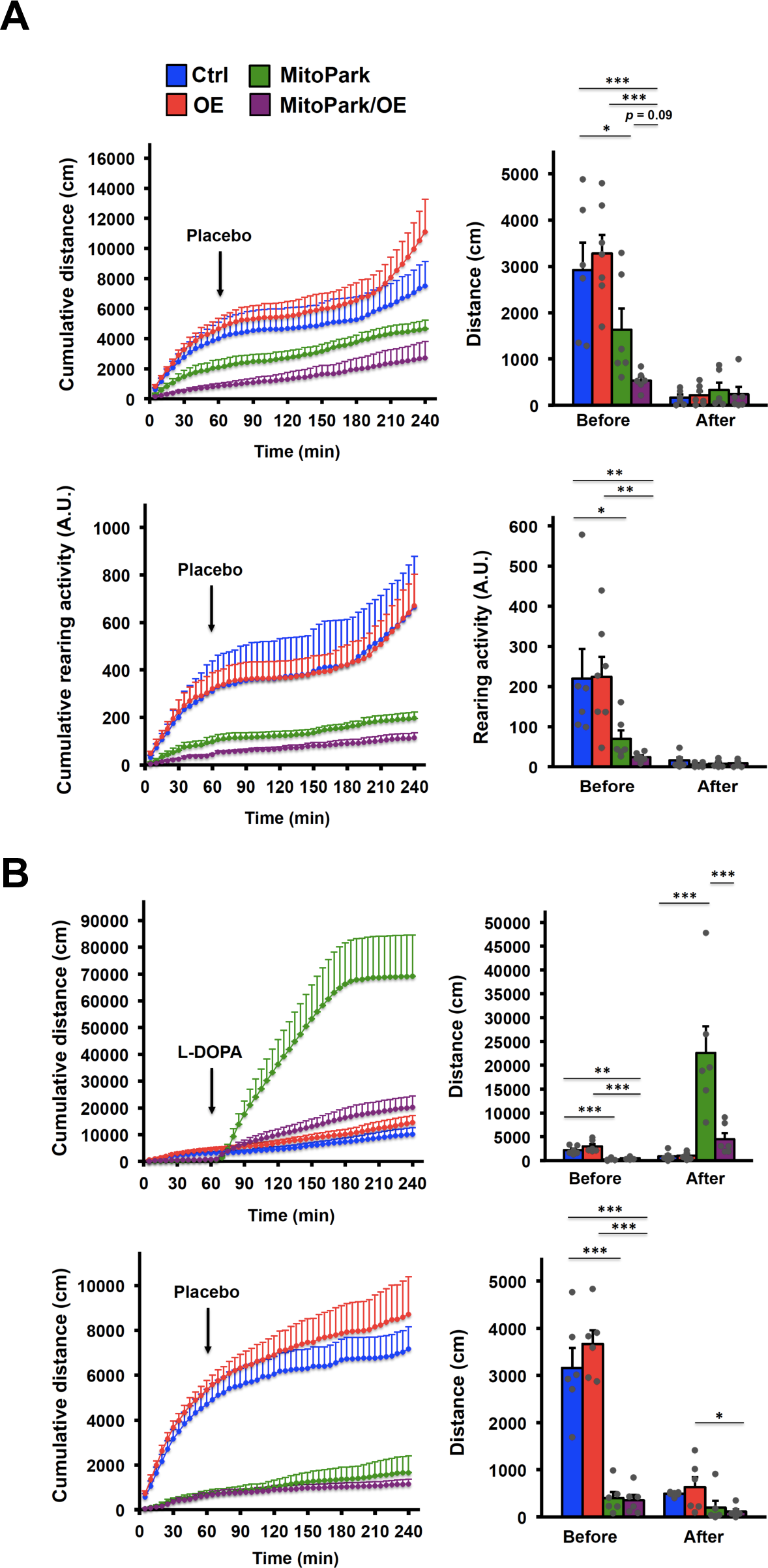

Supplement: S3 Fig — (A) Open-field assay recorded for 15- to 16-week-old Ctrl, OE, MitoPark, and MitoPark/OE mice treated with saline. n = 6-7/group. (B) Open-field assay recorded for 23- to 24-week-old mice treated with L-DOPA (20 mg/kg) (upper) or saline (lower). n = 6/group. (A-B) The arrows indicate reagent addition steps. Before, averaged activity from 0 to 30 minutes (pre-Saline/L-DOPA treatment); After, averaged activity from 75 to 105 minutes (post-Saline/L-DOPA treatment). *p < 0.05; **p < 0.01; ***p < 0.001. Mean ± SEM. One-way ANOVA Fisher’s LSD post hoc test. (TIF) [file pgen.1008868.s003.tif]

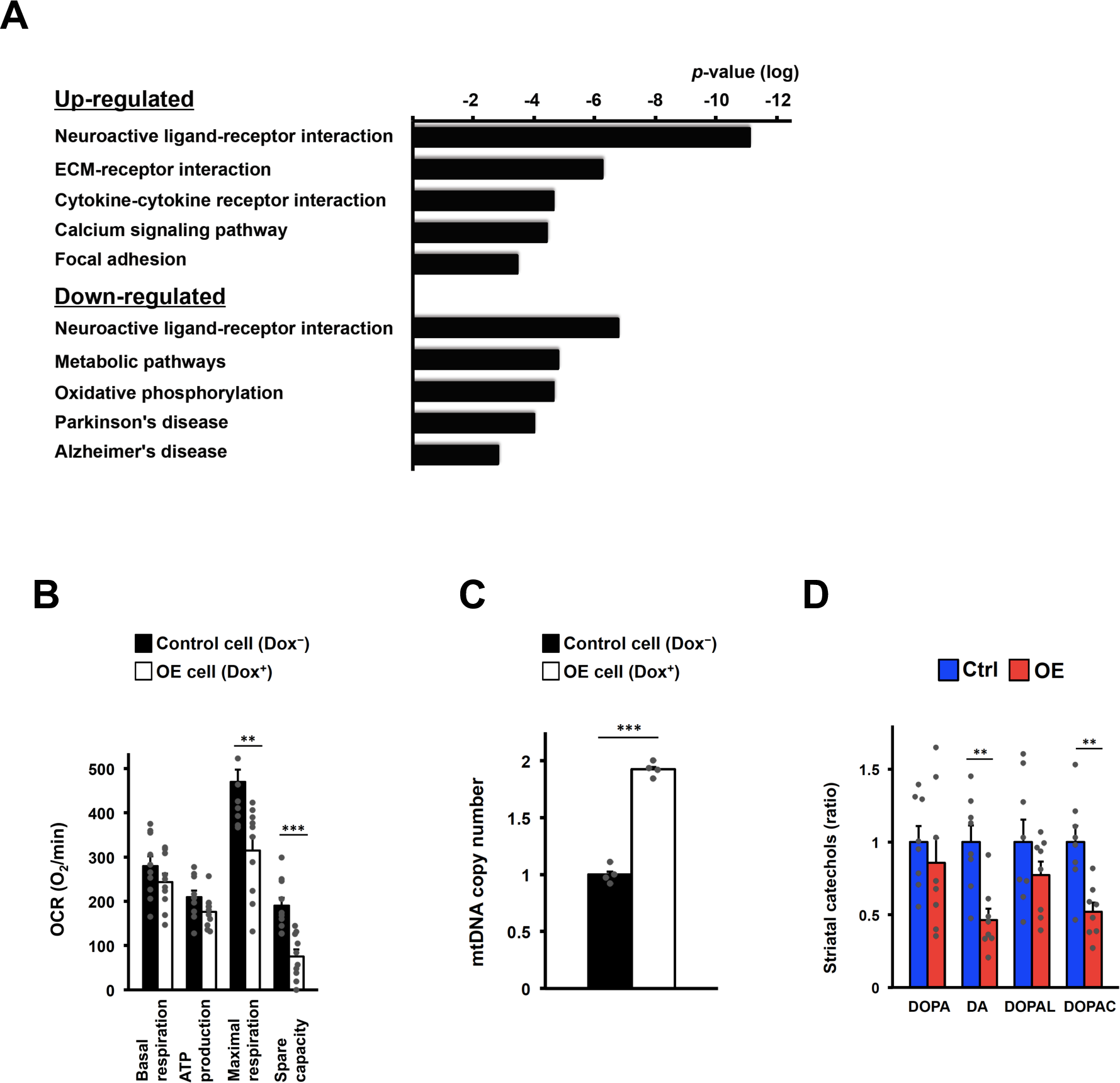

Supplement: S4 Fig — (A) Gene Ontology analysis of the transcriptional profiles from 2-month-old Ctrl and OE mice in the Kyoto Encyclopedia of Genes and Genomes. (B) Mitochondria oxygen consumption rates in the N27 rat DA neural cell line. n = 10/group. (C) Relative mitochondrial DNA (mtDNA) copy numbers in the N27 rat DA neural cell line. n = 4/group. (D) Relative total striatal catechols of 2-month-old Ctrl and OE mice. n = 8/group. (A-D) **p < 0.01; ***p < 0.001 compared to control (t-test). Mean ± SEM. (TIF) [file pgen.1008868.s004.tif]

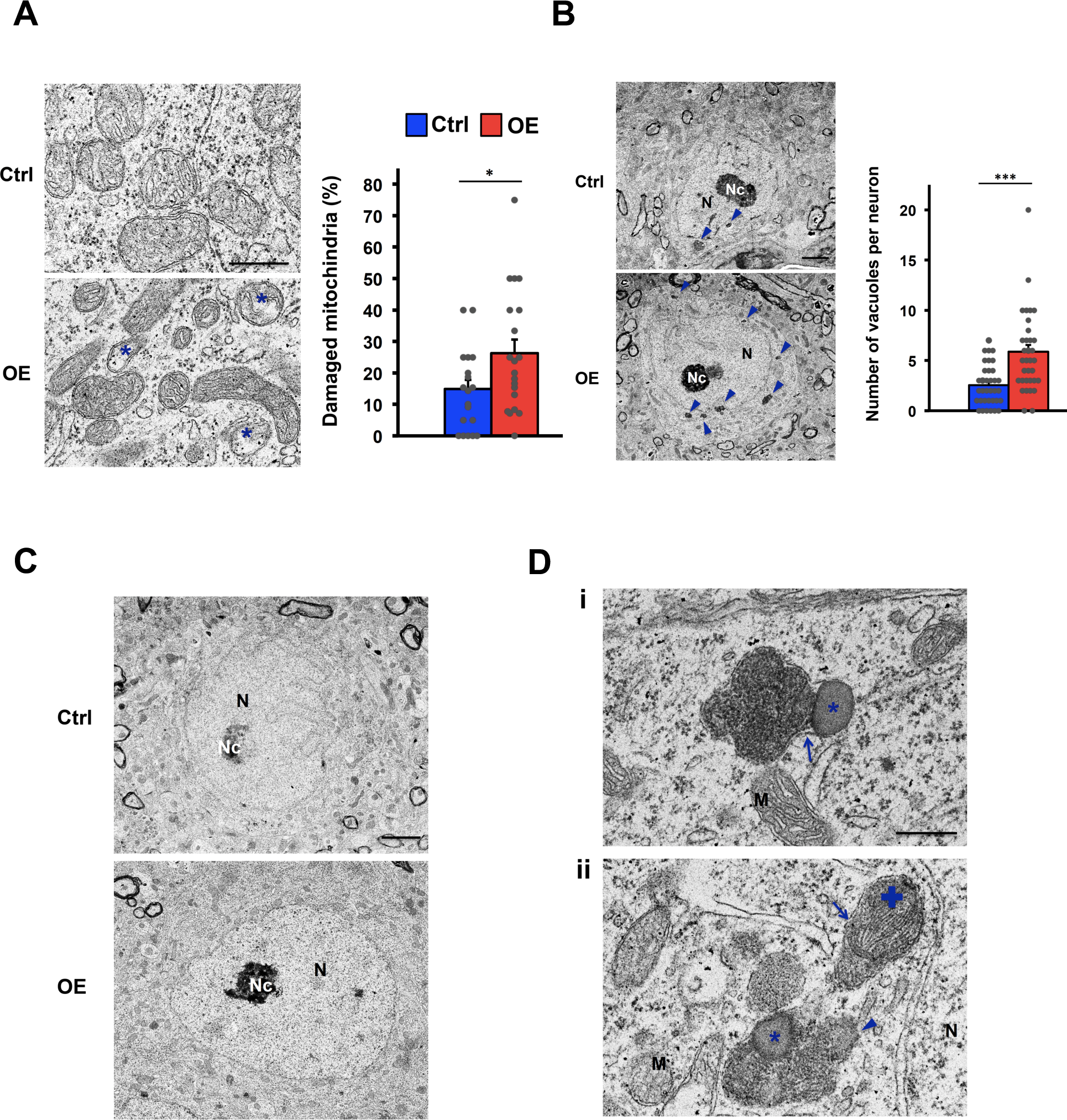

Supplement: S5 Fig — (A) Representative TEM images (left) and quantification (right) of damaged mitochondria (asterisks) in the SNpc of 10-month-old Ctrl and OE mice. n = 224 (Ctrl) and 212 (OE) mitochondria from 2 mice. Scale bar, 900 nm. (B) Representative TEM images (left) and quantification (right) of electron-dense vacuoles (arrowheads) in the SNpc of 10-month-old Ctrl and OE mice. n = 35 (Ctrl) and 34 (OE) neurons from 2 mice. Scale bar, 2 μm. (C) Representative TEM images of neurons in the SNpc of 3-week-old Ctrl and OE mice. Scale bar, 2 μm. (D) Higher-magnification views of various electron-dense vacuoles containing autophagosomes (arrows), autolysosome (arrowheads), mitochondria-like organelles (crosses) and lipofuscin granules (asterisks) in the SNpc of OE mice. Scale bar, 300 nm. (A-D) N, nucleus; Nc, nucleolus; M, mitochondria. *p < 0.05; ***p < 0.001 compared to control (t-test). Mean ± SEM. (TIF) [file pgen.1008868.s005.tif]

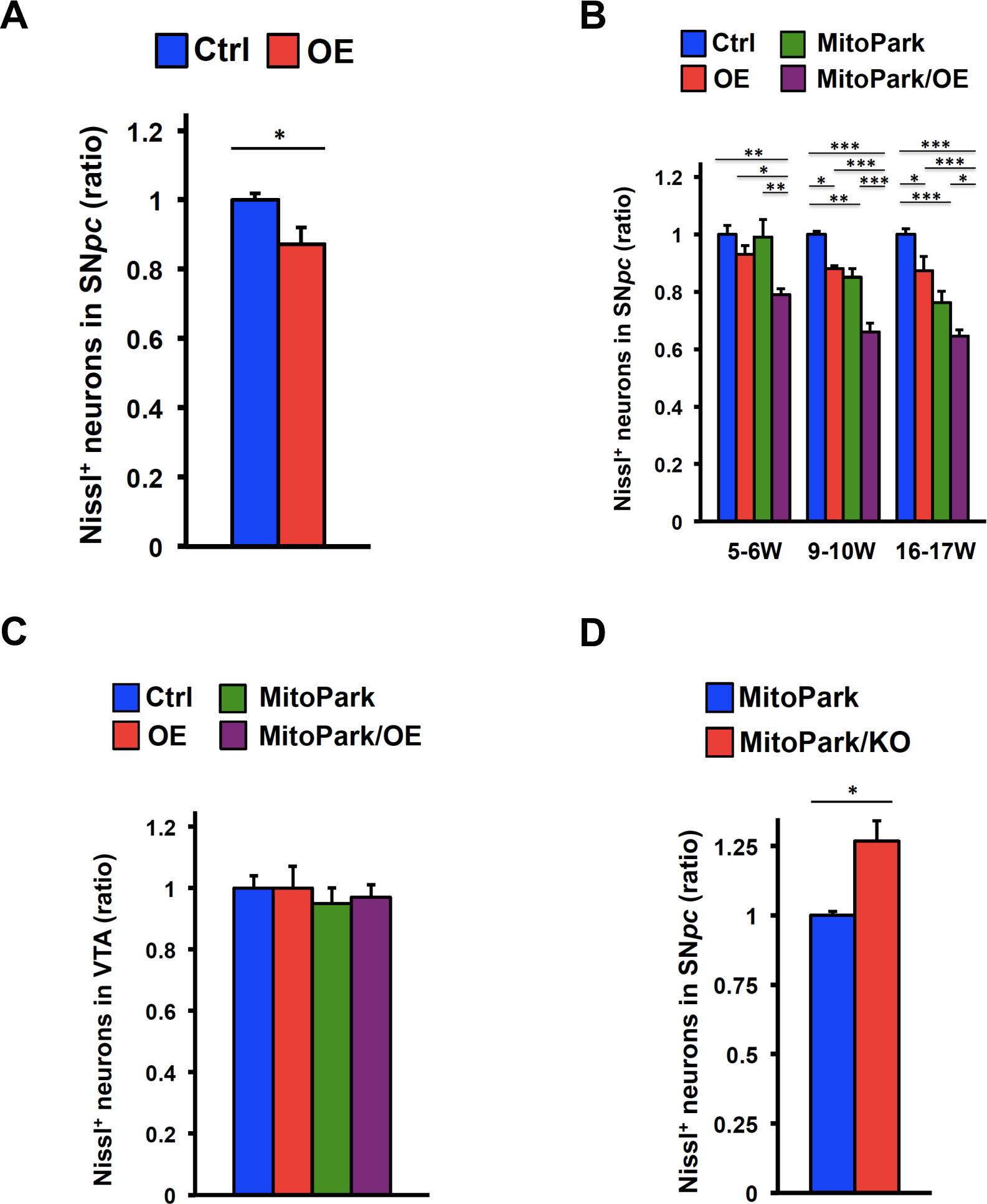

Supplement: S6 Fig — Numbers of total neurons were estimated by counting Nissl+ neurons in the SNpc or VTA, (A) refered to Fig 1H, (B) refered to Fig 2A, (C) refered to S2F Fig, and (D) refered to Fig 5A. *p < 0.05; **p < 0.01; ***p < 0.001. Mean ± SEM. t-test or One-way ANOVA Fisher’s LSD post hoc test. (TIF) [file pgen.1008868.s006.tif]
