# Supplementary material for: Elevated COUP-TFII expression in dopaminergic neurons accelerates the progression of Parkinson’s disease through mitochondrial dysfunction
Source: PLoS Genet. 2020 Jun 24;16(6):e1008868. doi: 10.1371/journal.pgen.1008868 (PMC7340320; doi:10.1371/journal.pgen.1008868)
Supplement: S2 Table — (PDF) [file pgen.1008868.s008.pdf]

Table S2. Primer sequence, related to Fig 1, 4, and 5.

Target	Sequence (5’ to 3’)
h-NR2F2	CGGAGGAACCTGAGCTACAC
	GGTACGAGTGGCAGTTGAGG
h-RPL32	GCACCAGTCAGACCGATATG
	ATGTTGGGCATCAAGATCTG
r-Rpl32	CAATGTTGGGCATCAGGATC
	TTAAGCGAAACTGGCGGAAAC
r-Ndufa13	TAATGAGGTGGAACCGGGAG
	GGTTCTCCCGAAGAATCTGC
r-Ndufaf4	ATGGCACCCAAGCACCCTTC
	GCACCGGATCTTTGGAATTG
r-Ndufs6	GGAGAAGATCACGCATACCG
	CGGTCCACCTCATTCACAGG
r-Sdha	GTGAGAGCAAGAAGGCATCC
	CCGAATGCAGCTCGCAAGCC
r-Cox6b1	GGCAGAACTACCTGGACTTC
	CACGCCGGTACCACTCACAC
r-Cox10	GTCACGGTTCAGCACTTGAG
	TGCTCAAGAAGAGGAGAAGC
r-mRps33	CATGGGCACCCTCCGTTTTC
	TTCCCCCGGAGCTTCTTTAG
r-mRps36	TTCCGCCACCAGGGTCGTGC
	TAGCGCAGCAGATCTCAAAG
r-mRpl4	CACCCCGACGTGTTTGCAAC
	GGTCTTGGTCTTAGCATAGC
r-mRpl34	TCGGTGATCCTGTTGAGTGG
	ATGTTGCTCGGCTGATACTC
r-Aldh1a1	CAATGAATGGCATGATTCAG
	GCCTTCACAGCTTTGTCAAC
r-Aldh1a2	AGGACGTCTGCTGGACAAGC
	CTCAGGGTTTTGATGACTCC
r-Aldh1a3	ATGTGAGGTGGAAGAAGGCG
	GCAACAACTGGCCACGACTC
r-Aldh2	CTGTCTTCACAAAGGACCTG
	CCCGACATCTTATAGCCACC
r-Aldh3a1	CATCCCTCAGTATATGGACC
	AACAATCTTCCCTACGGCTG
r-Aldh3b1	CAAACAGCAGCTGCAGGAGG
	TCATCCAAGACCGCAGGTTC
m-Ndufa13	GGCCTTGATCTTTGGCTACTG
	CACCCATCGTGTGGTATGGAA
m-Ndufaf4	CACCGGAGTCAGTATCCAGAA
	GGTTCAACTTTTACCGGCAAGG
m-sdha	GGAACACTCCAAAAACAGACCT
	CCACCACTGGGTATTGAGTAGAA
m-Cox6b1	ACTACCTGGACTTCCACCG
	ACCCATGACACGGGACAGA
m-Cox10	AGAAGAGCTATACAGGGATTGCC
	CTGTGTGACATACATGCGCTT
m-Ndufs6	GGGGAAAAGATCACGCATACC
	CAAAACGAACCCTCCTGTAGTC
m-mRps33	GAGTGCCCGGATCTTTGGTG
	AGCGCAAAATACGTGTTGTGA
m-mRps36	GACAAACCTAAACTCAGTGCCT
	AATCGGGGGACGTACTTCCTT
m-mRpl4	CGAGAGGTCCTCCGAGATG
	AGCCAGAGAGTCGAAGCGA

m-mRpl34	ATCGGTGGCCCTATTGAGTG
	GCTCGGCTGATACTCGTTTC
m-Aldh1a1	GAACCTATTGGAGTGTGTGG
	AGATGCCAGGTGAAGAGCCG
m-Aldh1a7	GCAACTGAGGAGGTCATCTG
	TCTGAAGCATCCATGGTGCG
m-Aldh3a1	AAGGCATCTCCAAGGCGCTG
	CCTCAGCCCAATCGGAGAGC
m-Aldh18a1	ACGCCTGGCATCTATTGTTG
	GCGACGGCTCCACTGGTCAC
m-Rpl32	TTAAGCGAAACTGGCGGAAAC
	TTGTTGCTCCCATAACCGATG
Mt-nd1 (DNA)	TGTTCCCAGAGGTTCAAATC
	GGAAGGCCATGGCAATTAAG
Gapdh (DNA)	CCCTGTGCATGTTTCCATAC
	AGAATGGGAAGCTGGTCATC
Me-COUP-TFII-5	GATATATTTCCCTGCTGTTGCG
	CTCGATACCCATGATGTTGTTG
Me-mCII-4	CCTCAAATCAACTAGCCCTG
	CTTCTGCTCCCCTGGCTGCG
H3Ac-CII-ChIP-0.5K-D	CGCGGACCACTTTCATGCTG
	TCAGGAGGACTAGGAGATCG
H3Ac-CII-ChIP-0K-U	CGAGTTGCCTCCTTTCTCCG
	GAGAGAGGAGGGCAGATCAC
H3Ac-CII-ChIP-1K-U	AGAACGGCCTTTATCCTCTG
	GGTGATGGCAAACCCTTCTC
H3Ac-CII-ChIP-2K-U	CTATCTGCCCAGATCTTTAG
	GAAAACATCCTTGGTAGCTG
